# Assessment of chemo-mechanical impacts of CO_2_ sequestration on the caprock formation in Farnsworth oil field, Texas

**DOI:** 10.1038/s41598-022-16990-x

**Published:** 2022-07-29

**Authors:** Benjamin Adu-Gyamfi, William Ampomah, Jiawei Tu, Qian Sun, Samuel Erzuah, Samuel Acheampong

**Affiliations:** 1 New Mexico Institute of Mining and Technology, 801 Leroy Place, Socorro, NM 87801 USA; 2 Petroleum Recovery Research Center, 801 Leroy Place, Socorro, NM 87801 USA; 3 School of Energy Resources, China University of Geosciences, 29 Xueyuan Rd., Beijing, China; 4 Kwame Nkrumah University of Science and Technology, Private Mail Bag, University Post Office, Kumasi, Ghana

**Keywords:** Chemical engineering, Biogeochemistry

## Abstract

This study evaluates the chemo-mechanical influence of injected CO_2_ on the Morrow B sandstone reservoir and the upper Morrow shale caprock utilizing data from the inverted 5-spot pattern centered on Well 13-10A within the Farnsworth unit (FWU). This study also seeks to evaluate the integrity of the caprock and the long-term CO_2_ storage capability of the FWU. The inverted 5-spot pattern was extracted from the field-scale model and tuned with the available field observed data before the modeling work. Two coupled numerical simulation models were utilized to continue the study. First, a coupled hydro-geochemical model was constructed to simulate the dissolution and precipitation of formation minerals by modeling three intra-aqueous and six mineral reactions. In addition, a coupled hydro-geomechanical model was constructed and employed to study the effects of stress changes on the caprock’s porosity, permeability, and ground displacement. The Mohr–Coulomb circle and failure envelope were used to determine caprock failure. In this work, the CO_2_-WAG injection is followed by the historical field-observed strategy. During the forecasting period, a Water Alternating Gas (WAG) injection ratio of 1:3 was utilized with a baseline bottom-hole pressure constraint of 5500 psi for 20 years. A post-injection period of 1000 years was simulated to monitor the CO_2_ plume and its effects on the CO_2_ storage reservoir and caprock integrity. The simulation results indicated that the impacts of the geochemical reactions on the porosity of the caprock were insignificant as it experienced a decrease of about 0.0003% at the end of the 1000-year post-injection monitoring. On the other hand, the maximum stress-induced porosity change was about a 1.4% increase, resulting in about 4% in permeability change. It was estimated that about 3.3% of the sequestered CO_2_ in the formation interacted with the caprock. Despite these petrophysical property alterations and CO_2_ interactions in the caprock, the caprock still maintained its elastic properties and was determined to be far from its failure.

## Introduction

This study is an evaluation of the chemo-mechanical impacts of injecting CO_2_ into the partially depleted Morrow B sandstone oil reservoir in the Farnsworth Unit (FWU) in Ochiltree County Texas. We used a series of simulation studies to evaluate caprock integrity and long-term storage capability. The evaluation of the relative contributions of various trapping mechanisms to overall CO_2_ storage was part of the interest.

The CO_2_ concentration in the atmosphere over the past centuries has risen dramatically, as has the exploitation of fossil-based energy resources increased^1,2^. The primary contributing factor to the rise in CO_2_ concentrations in the hydrocarbon utilization for transportation, electricity, home, and other industrial purposes^3,4^. There is a global consensus that increasing CO_2_ concentrations will disturb the earth’s climate, increase sea level, and damage sensitive ecosystems if nothing is done to curtail this problem^5,6^. According to Benson^7^, to avoid significant damage to the environment and ecosystems, the CO_2_ levels in the atmosphere need to be reduced and stabilized in the next couple of years. To achieve a reduction in the atmospheric CO_2_ emission, geologic storage, via the Carbon Capture and Storage (CCS) and Carbon Capture, Utilization, and Storage (CCUS) technologies, has been one of the promising methods for CO_2_ storage^8–13^. Nevertheless, the long-term storage and safety of the sequestered CO_2_ in the geological formations is critical since the injected CO_2_ could have the potential to escape from the target storage formation to shallower formations and eventually be released back into the atmosphere. Prior experiences gained from the injection of CO_2_ into matured oil reservoirs since the 1980s, according to Stevens et al.^14^, have presented researchers and the industry with preliminary assessments of the near-term effects and performance of CO_2_ injection into geologic formations. Also, this previous work is a stepping-stone for researchers to investigate the long-term effects of CO_2_ injection on geologic formations.

For geological sequestration of CO_2_ to be possible the following components must be found in a geological system; (a) a porous and permeable formation that will act as a storage “tank,” (b) an impermeable or low-permeability seal to serve as a barrier to CO_2_ flow, (c) secondary reservoir and seal to trap leaking CO_2_ in the case of primary seal failure^8,15^. Depleted oil and gas reservoirs are estimated to have about 400 to 900 Giga-tonnes of CO_2_ sequestration potential according to Bachu et al.^16^. Furthermore, these reservoirs have been found relatively suitable for storing CO_2_ as they have already established their ability to hold and safely store fluid for millions of years^16–18^.

The trapping mechanisms associated with the geologic injection of CO_2_ are structural, residual, solubility, and mineral trapping^19,20^. An effective CO_2_ sequestration operation requires monitoring CO_2_ plume movement in the reservoir and any leakage to the atmosphere for several thousands of years^21^. However, CO_2_ movement is relatively complex since it entails the impacts of pore fluid dynamics, formation of minerals and lithology, geochemical effects such as mineral dissolution and precipitation, and stress changes over time^8^. Ultimately, the caprock must be able to withstand the chemical and physical property changes caused by interactions of the CO_2_, brine, and formation minerals and the changes in the stress field during and after the injection of CO_2_. As the years go by, the storage system may be subject to tension, compression, alteration of mineral composition, and/or formation of fractures or faults due to the changes in physical, chemical, and stress patterns^8^. As a result, the strength and integrity of the caprock may be compromised, leading to CO_2_ leakage. CO_2_ leakages as a result of geologic sequestration could be potentially associated with the risk of reactivation of faults, induced shear failure, fracturing, and leakage via injection or production wells^8–10,22^.

Frash et al.^23^ investigated the fracturing and fluid flow in samples taken from the carbonate-rich Marcellus shale in Bedford, Pennsylvania. In these experiments, they measured fracture geometry, induced permeability, displacement, and stresses to investigate the effects of fracturing and fluid flow in a potential CO_2_ leakage within the Marcellus caprock. The stress distribution encountered in the experiments with verified by a numerical simulation. The experiment results indicate that the induced permeability due to fracturing depended on the duration of flow, stresses at which fractures were created, and the magnitude of the shearing displacement. In another experiment, Okamoto et al.^24^ used samples from the caprock of the Nagaoka injection test site and treated them with supercritical CO_2_. The authors found that these particular samples resulted in less than 1% change in permeability and a less significant increase in porosity.

Using the Eclipse reservoir simulator, Lindeberg^25^ modeled the leakage of CO_2_ through aquifers. The author proved that the distribution of CO_2_ after being injected and its subsequent escape was dictated by gravity forces and the horizontal permeability of the caprock/reservoir interface. Saripalli and McGrail^10^ used a semi-analytical approach to model a hypothetical case of CO_2_ injection for geologic sequestration of CO_2_. In modeling the CO_2_ leakage through the caprock, they considered two significant pathways: vertical migration of free gas through fractures and flow through permeable water-saturated caprock due to buoyancy force.

Rutqvist et al.^22^, using a hypothetical model system consisting of a caprock and aquifer, analyzed the spread of CO_2_ plume, ground displacement, stress changes, mechanical failure, and stress-induced permeability changes. In their work, they modeled stress-induced porosity and permeability as follows:

1 $$\phi = \left( {\phi_{o} - \phi_{r} } \right)\exp \left( {5 * 10^{ - 8 } * \sigma_{M}^{^{\prime}} } \right) + \phi_{r}$$

2 $$k = k_{o} exp\left[ {22.2 x\left( {\frac{\phi }{{\phi_{o} }} - 1} \right)} \right]$$

where $\sigma_{M}^{^{\prime}}$ is mean effective stress, $\phi_{o}$ is the porosity at zero stress, $k_{o}$ is permeability at zero stress, and $\phi_{r}$ is the residual porosity at high stress. Equations (1) and (2) were determined by laboratory experiments conducted by Davis et al.^26^. Rutqvist et al.^22^ found out that the lower part of the caprock or the reservoir/caprock interface faces the highest risk of rock failure due to significant hydromechanical changes experienced due to a high effective mean stress reduction. As such, the tendency of the lower layers of the caprock to hydraulically fracture was very high in their study.

The relationships between injection pressure, total displacement, and effective stress that affect the integrity of caprock during CO_2_ injection into an oil reservoir were evaluated by the works of Karimnezhad et al.^27^. This work used a 3D numerical finite element reservoir model to assess the geomechanical effects and the risks of caprock failure associated with CO_2_ injection. The model had a cylindrical geometry with a diameter of 10 km with the reservoir and caprock thickness of 276 m and 63 m, respectively. Supercritical CO_2_ was injected at a 0.01 Mt/year rate for ten years in the single vertical injection well placed in the model's center. In addition, Karimnezhad et al.^27^ followed the Mohr–Coulomb rock failure criterion (Eq. 3) to determine whether or not the rock had undergone any potential shear failure.

3 $$\tau = c + \left( {\sigma_{n} - P} \right)\tan \varphi$$

where $\tau$ is the shear stress, $\varphi$ is the internal friction angle, $c$ is cohesion, $\sigma_{n}$ is the normal stress, and $P$ is the pore pressure.

The tensile failure was evaluated using the following tensile failure criterion in Eq. (4).

4 $$\sigma_{T} + \sigma_{3}^{^{\prime}} \le 0$$

$\sigma_{T}$ denotes rock matrix tensile strength as $\sigma_{3}^{^{\prime}}$ denotes the minimum effective principal stress.

Upon analyzing the results, Karimnezhad et al.^27^ noticed that the induced geomechanical changes were greatest during the initial CO_2_ injection and near the injection wellbore; however, the geomechanical changes generally diminish gradually with time and distance away from the injection site. Therefore, the most critical zone in the caprock where the most significant risks of failure could occur was determined to be the zone with the maximum uplift. The Mohr failure envelope constructed from their results showed no shear failure. However, the sensitivity study conducted on variable injection rates indicated the caprock undergoing tensile failure at an injection rate of 0.28 Mt per year or higher. This assessment did not consider any geochemical changes.

**Figure 1:**
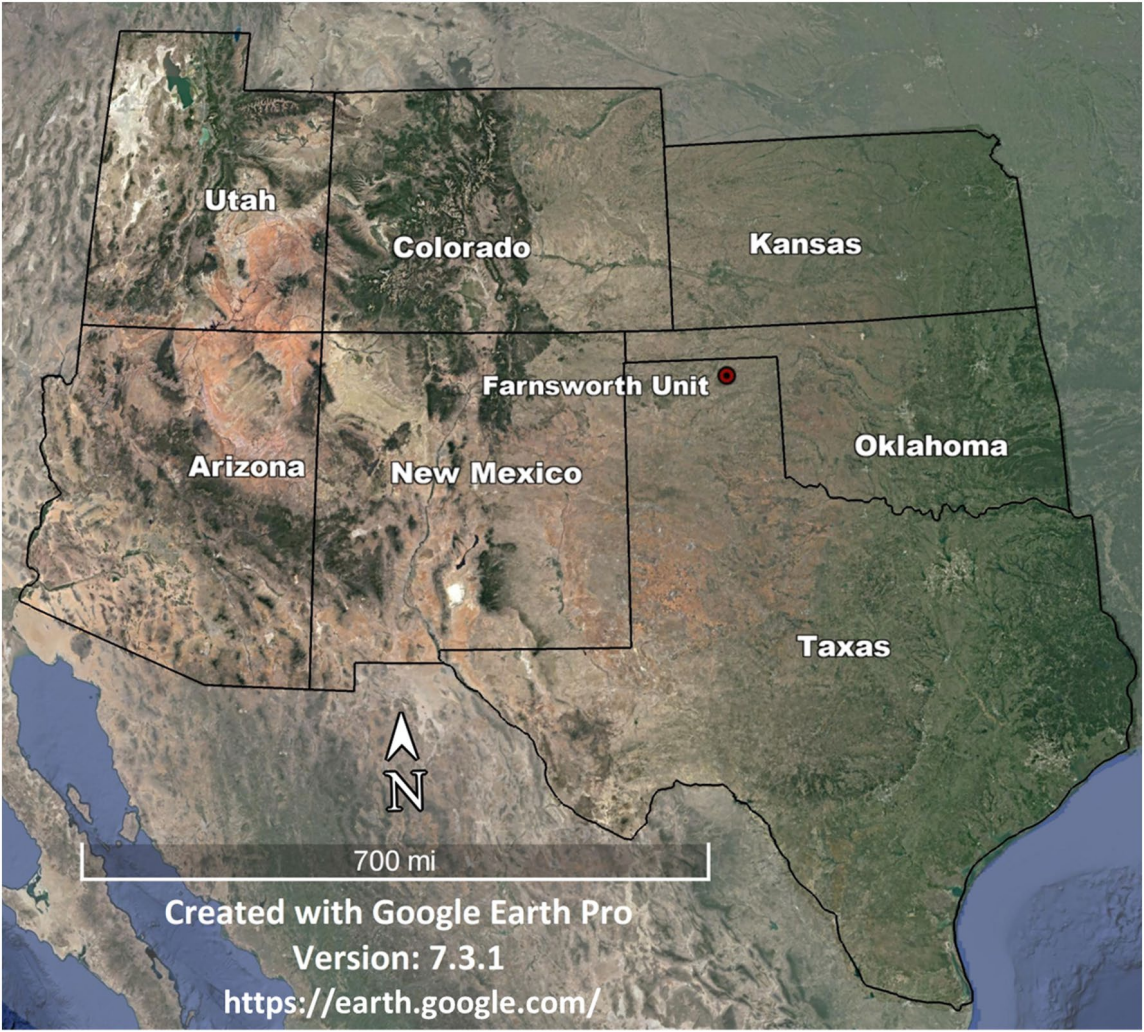
Location of FWU indicated by the red circle^33^.

The Southwest Regional Partnership (SWP) was established by the United States Department of Energy (DOE) in 2003 and tasked to study carbon management strategies^28,29^. The study in this paper uses data from one of the SWP’s field locations the Farnsworth Unit (FWU), located in Ochiltree County in Texas (Fig. 1). The FWU has its primary reservoir as the Upper Pennsylvanian Morrow B sandstone, which lies between the Morrowan-aged shales at a depth extending from 7550 to 7950 ft^30^. The formation was deposited in an incised valley and had an average dip angle of less than 1^o^^31,32^. The upper Morrow reservoir at FWU contains multiple sandstone packages separated by mudstone^32^. The main caprocks of FWU are considered to be the Thirteen Finger limestone and the upper Morrow shale^32^. This study focuses on the Morrow B sandstone reservoir and the upper Morrow shale caprock.

Considering the CO_2_ EOR operation already underway in FWU, it was deemed useful to evaluate the chemo-mechanical effects on the reservoir-caprock interface to determine the long-term storage capability of FWU. The increase in pressure due to the ongoing water-alternate-gas operation (WAG) might cause the reservoir and caprock to experience a reduction in effective stress leading to stress-induced alterations of permeability and porosity, potentially causing CO_2_ migration across the reservoir-caprock interface. In addition, chemical reactions during CO_2_ injection could also reduce the pH of the in-situ fluid, making it more acidic and leading to the dissolution of minerals, again, causing permeability and porosity alterations. In this study, the chemo-mechanical impacts on the Morrow B sandstone—upper Morrow shale caprock interface are evaluated to ascertain the integrity of the caprock of FWU.

## Methodology

**Figure 2:**
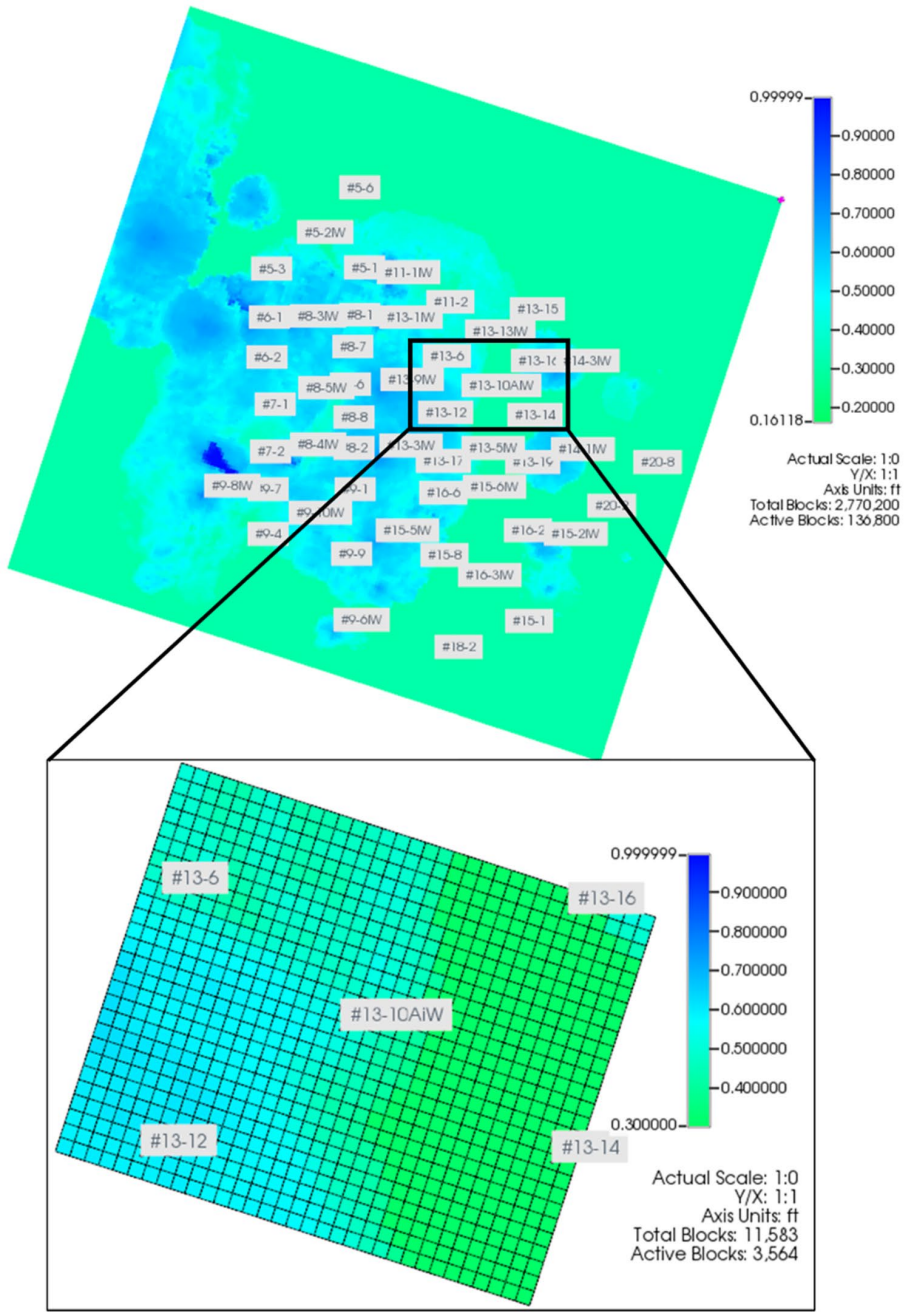
Extracted sector model (13-10A inverted 5-spot pattern) from field-scale reservoir model.

**Figure 3:**
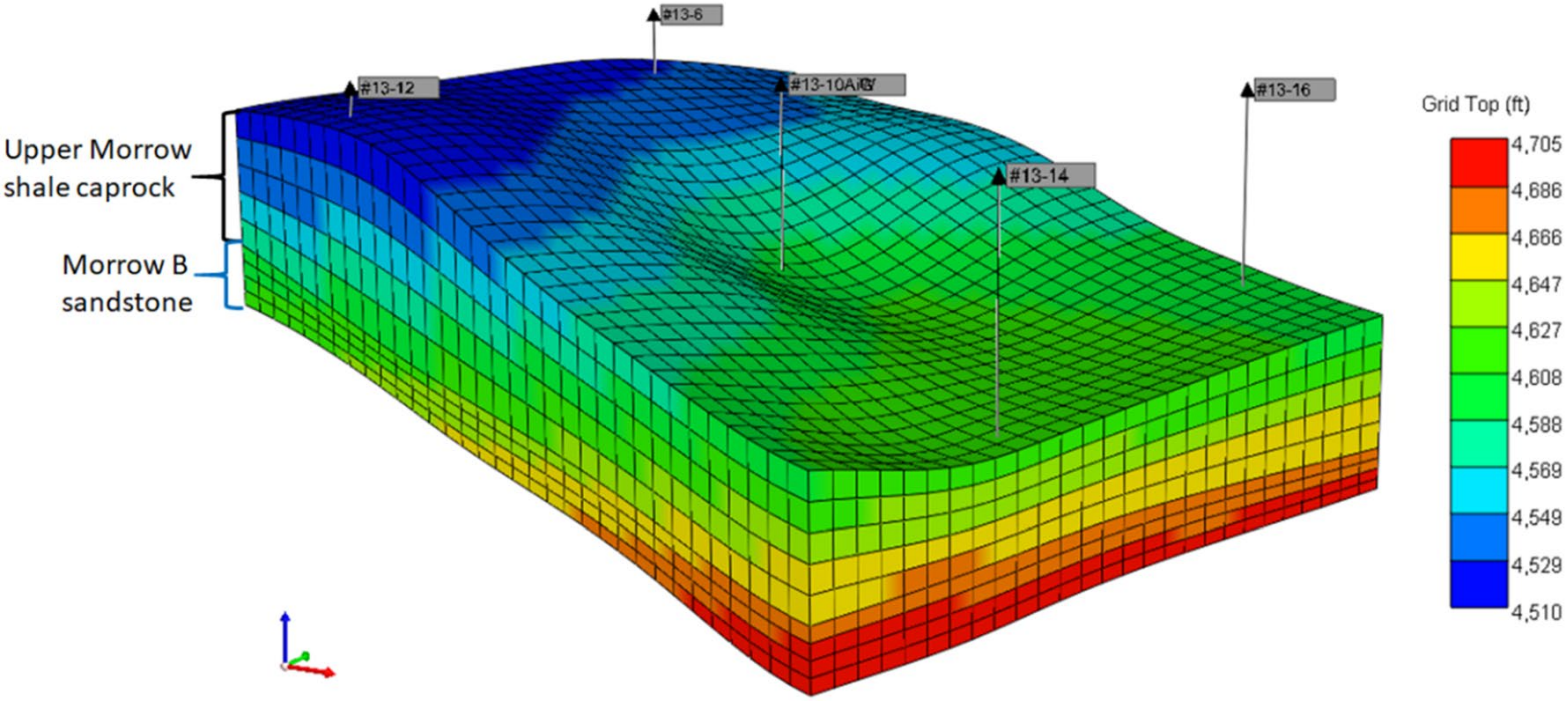
3D view of the 13-10A inverted 5-spot pattern consisting of the Morrow B sandstone and the upper Morrow shale. Layer 1 is the top layer of the caprock and numbers increase with depth through to Layer 9 indicated as the bottom layer of the reservoir.

**Figure 4:**
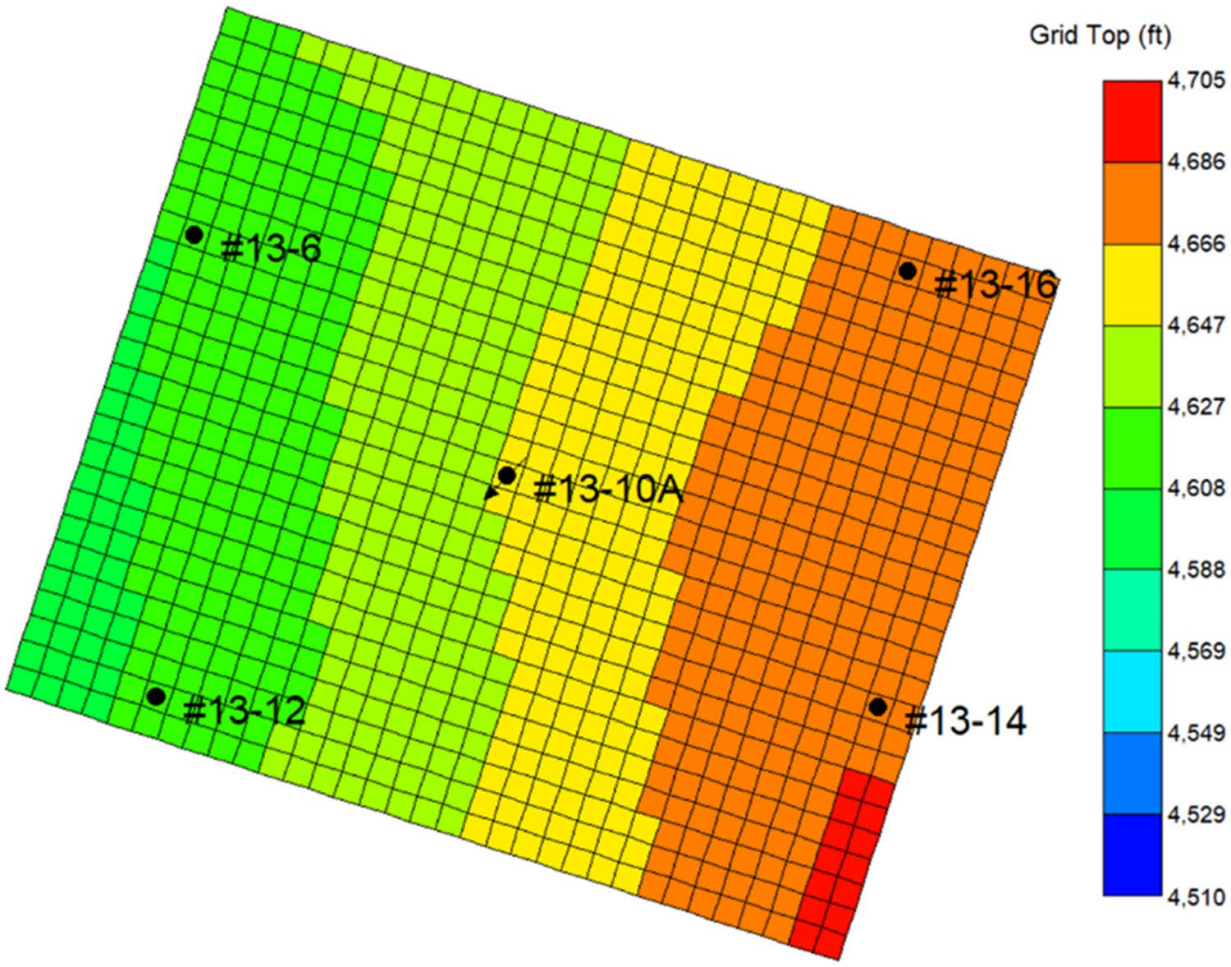
2D areal view of the 13-10A inverted 5-spot pattern, showing the production wells and the injection well.

Multiphase compositional simulator CMG-GEM distributed by Computer Modeling Group (CMG) LTD. is used to construct the model and compute the results presented in this paper. The model used for this study is an inverted 5-spot section model extracted from the recent version^34,35^ of a full-field FWU geological model, and the aerial view of both models is shown in Fig. 2. As shown in Figs. 3 and 4, the section is a 3300 ft by 2700 ft area that was comprised of a 100 ft by 100 ft uniform areal dimension of grid cells. The reservoir consists of four layers and the caprock consists of five layers. The baseline mineralogy and volume fractions for reservoir and caprock were based on the ELAN analysis performed on two science wells in the 13-10A pattern. Boundary conditions were defined with the assumption that the injection zone and confining zones are continuous throughout the region. No-flow boundaries were assigned to the uppermost Morrow shale and lowermost Morrow B sandstone formations in the model. The lateral boundaries were assigned with a close boundary as the inverted 5-spot follows the mirror image rule. This assumption was also verified during the history matching. Table 1 presents the volume fractions used to initialize the model. The original full-field model had undergone primary and secondary recovery phase history matching^36^. The extracted sector model was then history matched in the tertiary recovery phase. After the history matching process, this study created a coupled hydro-geochemical model and a coupled hydro-geomechanical model to evaluate the effects of CO_2_ injection on the reservoir–caprock interface. Both models were constructed to account for structural trapping, residual gas trapping, CO_2_ solubility in water, and CO_2_ solubility in oil. The forecasting strategy used was a target of 2 MM SCF/day CO_2_ injection rate with a baseline BHP constraint of 5500 psi. The water injection rate was set at 900 STB/day with a baseline BHP constraint of 5500 psi.

**Table 1:** Volume fraction of FWU minerals determine by ELAN analysis.

Mineral	Volume fraction					
	Calcite (%)	Quartz (%)	Illite (%)	Kaolinite (%)	Pyrite (%)	Chamosite (%)
Morrow B	0.09	70.31	3.54	1.35	0.53	8.60
Upper morrow shale	0.70	44.48	8.46	18.40	0.49	23.69

### History matching process prior to CO_2_-EOR operations

After extracting the 5-spot sector model, a compositional numerical simulation baseline model was constructed, and a closed boundary was applied to it. A sensitivity analysis was performed on selected uncertain parameters to determine the most influential parameters. These uncertain parameters comprised the relative permeability endpoints, critical saturation endpoints, and Corey correlation parameters of the five pairs of relative permeability curves data. Also included were the directional permeability multipliers. Each of the selected parameters was assigned a value based on the baseline model. However, a range of values was assigned based on the knowledge gained from the characterization and experimental work prior to this study. A polynomial response surface methodology was utilized. The methodology uses a proxy model to evaluate the relations among the selected parameters and selected objective functions (oil, water, and gas production rates; and the gas and water injection rates)^37^. A total of 238 simulation runs were completed to train and verify the proxy model. Almost all the parameters significantly influenced one or more objective functions. Therefore, all the parameters were included in the history match processes.

**Figure 5:**
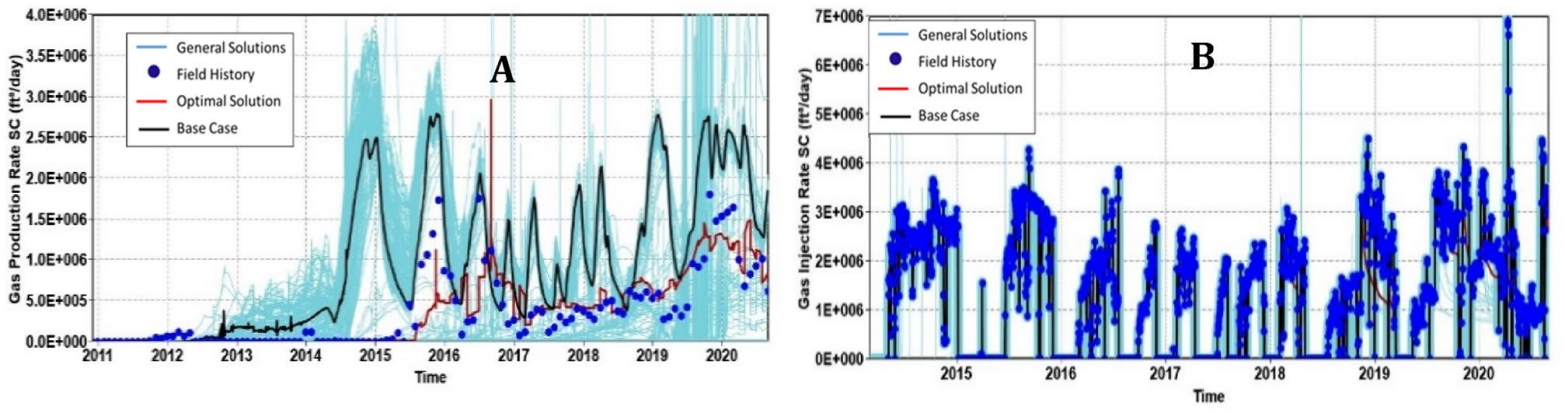
History matching performances on the (**A**) gas production, (**B**) gas injection rate.

Particle Swarm Optimization (PSO) was employed in the history matching process to minimize the errors between the simulation results and the actual field measurement. The PSO method attained a history matching error of 8.81% after 355 simulation runs. The results of the history matching are shown in Fig. 5.

### Trapping mechanisms

#### Residual trapping

The trapping of CO_2_ as residual gas was modeled by relative permeability hysteresis. Residual gas trapping involves trapping the nonwetting phase (CO_2_) due to capillary and wettability effects by shifting from drainage to imbibition curves^38–40^. Spiteri et al.^41^ described several gas trapping models that were used for estimating residual gas saturations in their work. The three-phase water-alternating-gas hysteresis (3PWAG) model was utilized in modeling residual gas trapping, according to Larsen et al.^42^. This 3PWAG model is described by Eqs.(5–8). The trapped gas saturation, $Sgr$, is defined by Eq. (5).

5 $$S_{gr} = S_{gcrit} + \frac{{\left( {Sgm - Sgcrit} \right)}}{{\left( {1 + C\left( {Sgm - Sgcrit} \right)} \right)}}$$

The gas relative permeabilities, $Krg$, on the drainage curve to the imbibition scanning curve were modeled by:

6 $$K_{rg} \left( {Sg} \right) = K_{rg}^{drain} \left( {Sgf} \right)$$

The free gas saturation, $S_{gf}$, is calculated as:

7 $$S_{gf} = { }S_{gcrit} + \frac{1}{2}\left\{ {\left( {S_{g} - S_{gr} } \right) + \sqrt {\left( {S_{g} - S_{gr} } \right)^{2} + \frac{4}{C}\left( {S_{g} - S_{gr} } \right)} } \right\}$$

A secondary drainage curve is estimated on the condition that the gas saturation decreases once again. The estimated secondary drainage curve is as follows:

8 $$K_{rg}^{drain} = \left[ {K_{rg}^{input} - K_{rg}^{input} \left( {S_{g}^{start} } \right)} \right]\left[ {\frac{{S_{wcon} }}{{S_{w}^{start} }}} \right]^{\alpha } + K_{rg}^{imb} \left( {S_{g}^{start} } \right)$$

#### Solubility trapping

To account for solubility trapping, CO_2_ dissolution in oil is modeled by injecting and maintaining the reservoir pressure above the CO_2_-oil minimum miscibility pressure of 4009 psia, as determined by Gunda et al.^43^. CO_2_ dissolution in brine was based on the theory that aqueous and gaseous phases are in thermodynamic equilibrium^41^. Therefore, the fugacities of the CO_2_ components in the aqueous and gaseous phases are the same and are represented by Eq. (9) as:

9 $$f_{co2,g} - f_{co2,aq} = 0$$

However, the fugacity of CO_2_ in the gaseous phase for this study was computed from equations of state (EOS), and that of CO_2_ in the aqueous phase was calculated from Henry’s law as follows:

10 $$f_{co2,aq} = H_{co2,aq} \cdot y_{co2,aq}$$

where $H_{co2,aq}$ is Henry’s law constant, and it depends on pressure, salinity, and temperature.

### Coupled hydro-geochemical modeling

CO_2_ injection into a mature oil reservoir triggers chemical interactions with the in-situ formation fluids and with the formation minerals. The chemical interactions between the injected CO_2_ and the in-situ formation brine represent the intra-aqueous reactions. Therefore, three intra-aqueous reactions were modeled and detailed in Table 2. Also, the interactions of CO_2_ and the formation minerals represent the geochemical (mineral) reactions. Table 3 details the mineral reactions and their associated reaction parameters. These intra-aqueous and mineral reactions are responsible for mineral precipitation and dissolution reactions. These in turn influence the porosity and permeability properties and determine if CO_2_ would be trapped as a carbonate mineral. The mineral precipitation and dissolution reactions were governed by Eqs. (11–13) according to Nghiem et al.^44^ and Nghiem et al.^45^.

11 $$r_{\theta } = \hat{A}_{\theta } k_{\theta } \left( {1 - \frac{{Q_{\theta } }}{{K_{eq,\theta } }}} \right), \theta = 1,2, \ldots ., R_{mn}$$

12 $$Q_{\theta } = \mathop \prod \limits_{k = 1}^{{n_{aq} }} a_{k}^{{v_{k\theta } }}$$

13 $$\frac{{Q_{\theta } }}{{K_{eq,\theta } }} = saturation index$$

**Table 2:** Aqueous reactions with their LOG chemical equilibrium constants.

Aqueous reactions	LOG-CHEM-EQUIL-CONST for the aqueous reaction ($Log K_{eq,\alpha }$)
1. CO_2(aq)_ + H_2_O ⇌ H^+^ + HCO_3_^-^	− 6.3445
2. H^+^ + OH^-^ ⇌ H_2_O	12.6762
3. H^+^ + CO_3_^2-^ ⇌ HCO_3_^-^	10.2084

**Table 3:** Mineral reactions and reaction parameters.

Mineral reactions	LOG-CHEM-EQUIL-CONST for the aqueous reaction ($Log K_{eq,\theta }$)	Reactive surface area (m^2^/m^3^)	Rate constant at 25 °C (mol/m^2^s)	Activation energy (J/mol)
1. Quartz ⇌ SiO_2 (aq)_	− 3.3223	2650	− 11.3	41,870
2. Calcite + H^+^ ⇌ Ca^2+^ + HCO_3_^−^	1.0056	2989.25	− 5.81	23,500
3. Illite + 8H^+^ ⇌ 5H_2_O + 0.25Mg^2+^ + 0.6 K^+^ + 2.3Al^3+^ + 3.5SiO_2(aq)_	5.4416	27,500	− 12.78	35,000
4. Kaolinite + 6H^+^ ⇌ 5H_2_O + 2Al^3+^ + 2SiO_2(aq)_	3.7698	28,262	− 13.18	22,200
5. Chamosite-7A + 10H^+^ ⇌ 7H_2_O + 2Al^3+^ + 2Fe^2+^ + SiO_2(aq)_	2.4533	2710	− 11	41,870
6. Pyrite + H_2_O ⇌ 0.25H^+^ + 0.25SO_4_^2−^ + Fe^2+^ + 1.75HS^−^	− 2.2225	2710	− 8.8	41,870

### Coupled hydro-geomechanical modeling

A coupled hydro-geomechanical model was employed to examine the effect of the geomechanical response at the reservoir-caprock interface due to stress changes induced by CO_2_ injection. The model coupled hydrodynamics and geomechanics in a two-way coupling manner. The two-way coupling requires that the hydrodynamic simulator computes the reservoir pressure, temperature, saturation, porosity, and permeability. Then the computed pressure and temperature are passed to the geomechanics simulator, which computes deformation and stress changes. Finally, the porosity coefficients are computed and sent back to the hydrodynamic simulator. A detailed workflow and description for the two-way coupling approach, also known as an iterative coupled approach, can be found in literature^46–48^. The stress-induced reservoir porosity formulation is shown with Eqs. (14) to (19). In addition, the mechanical rock properties for the reservoir and caprock are detailed in Table 4.

14 $$\phi_{res}^{n + 1} = \phi_{res}^{n} + \left( {a + c \cdot d} \right)\left( {P - P^{n} } \right) + \left( {b + c \cdot e} \right)\left( {T - T^{n} } \right)$$

where

15 $$a = \frac{1}{{V_{b}^{o} }}\left( {\frac{{dV_{p} }}{dp} + V_{b} \alpha_{b} c_{b} \frac{{d\sigma_{m} }}{dp} - V_{b} \beta \frac{dT}{{dp}}} \right)$$

16 $$b = \frac{{V_{p} }}{{V_{b}^{o} }}\beta$$

17 $$c = - \frac{{V_{b} }}{{V_{b}^{o} }}\alpha_{b} c_{b}$$

18 $$d = \Gamma \left( {\frac{2}{9}\frac{E}{{\left( {1 - v} \right)}}\alpha_{b} c_{b} } \right)$$

19 $$e = \Gamma \left( {\frac{2}{9}\frac{E}{{\left( {1 - v} \right)}}\beta } \right)$$

**Table 4:** Mechanical rock properties of Morrow B sandstone and upper Morrow shale.

Parameter	Upper morrow shale	Morrow B sandstone
Young’s modulus	1.03 Mpsi	2.89 Mpsi
Poisson’s ratio	0.369	0.227
Cohesion	3131 psi	1746 psi
Internal friction angle	24.16 deg	40.77 deg

The geomechanical effect on permeability is based on the empirical modeling work presented by Li et al.^49^ and Touhidi-Baghini^50^. A matrix permeability multiplier was applied to update the permeability based on the volumetric strain and an experimentally determined dimensionless parameter ($T_{n1}$) as shown in Eqs. (20) and (21). Equation (20) applies to all three directional permeabilities.

20 $$\frac{k}{{k_{o} }} = \exp \left( {T_{n1} \cdot { }\varepsilon_{v} } \right)$$

where

21 $$T_{n1} = \left[ {\frac{{3\left( {1 - \phi_{o} } \right) + 2\phi_{o} }}{{\phi_{o} }}} \right]$$

**Figure 6:**
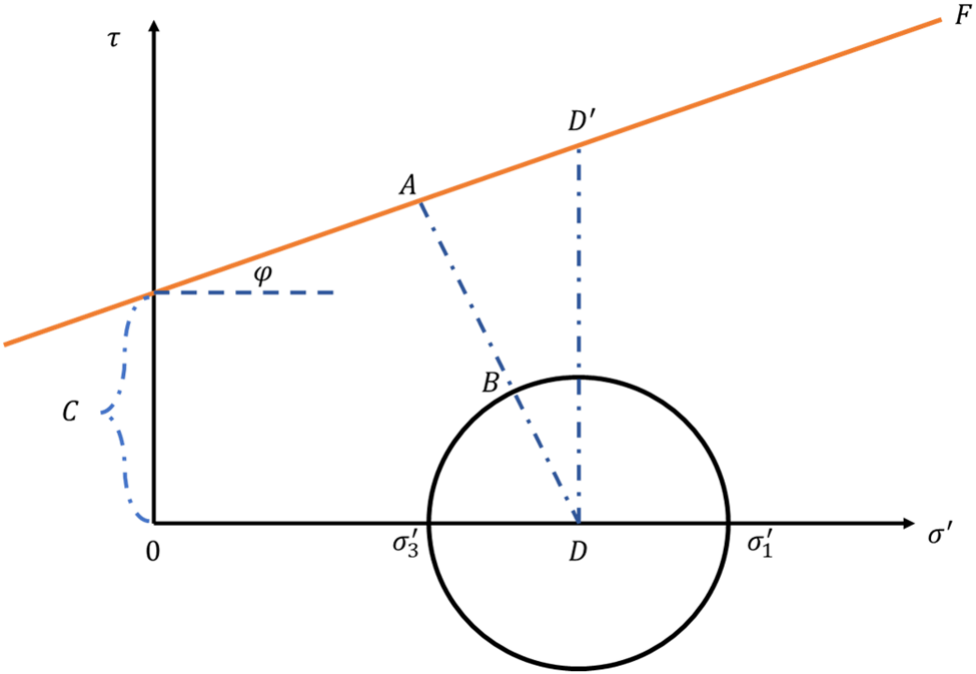
Mohr–Coulomb circle and failure line.

This study utilized the Mohr–Coulomb constitutive model to define the failure criteria for the caprock. The main rock failure modeled is the shear failure. The shear failure was also expressed as a shear safety factor (SF) determined considering the Mohr–Coulomb circle and the failure line in Fig. 6. The shear safety factor was estimated by Eqs. (22) to (26). A shear safety factor greater than zero indicates that the stresses are below the failure line, or the rock is elastic and safe from failure. However, if the shear factor is zero (the mohr circle is tangent to the failure line) or close to zero, the rock had failed or would be failing soon, respectively.

22 $$SF = 1 - \left( {minimum of \left[ {1, \frac{DB}{{DA}}} \right]} \right)$$

where

23 $$DB = \frac{{\left( {\sigma_{1}^{^{\prime}} - \sigma_{3}^{^{\prime}} } \right)}}{2}$$

24 $$DD^{\prime} = \frac{{\left( {\sigma_{1}^{^{\prime}} + \sigma_{3}^{^{\prime}} } \right)}}{2} \cdot \tan \left( \varphi \right) + C$$

25 $$DA = {\text{cos}}\left( \varphi \right) \cdot DD^{\prime}$$

26 $$DA = \cos \left( \varphi \right) \cdot \left[ {\frac{{\left( {\sigma_{1}^{^{\prime}} + \sigma_{3}^{^{\prime}} } \right)}}{2} \cdot \tan \left( \varphi \right) + C} \right]$$

$\sigma_{1}^{^{\prime}}$ Maximum effective stress, $\sigma_{3}^{^{\prime}}$ Minimum effective stress, $F$ Failure line and constructed by considering Eq. (3) ($\tau = c + \sigma^{\prime} \cdot \tan \varphi$).

### Pressure sensitivity analysis

A pressure sensitivity analysis was conducted to ascertain how pressure contributes to the shear failure of the caprock and to determine at what pressure the caprock would fail. The sensitivity study considered injecting CO_2_ over the baseline BHP of 5500 psi. Specifically, CO_2_ was injected at a BHP of 7000 psi, 7500 psi, and 8000 psi for an additional five years beyond the baseline forecasting period.

### Unit conversion factors

**Table:** 

kPa	=	6.8948 * psi
Kg	=	1000 * Metric Ton
m^3^	=	0.159 * bbl
m	=	0.3048 * ft
m^2^	=	0.0929 * ft^2^
m^3^	=	0.02832 * ft^3^

## Results and discussion

### Geochemical response on the caprock due to CO_2_ injection

**Figure 7:**
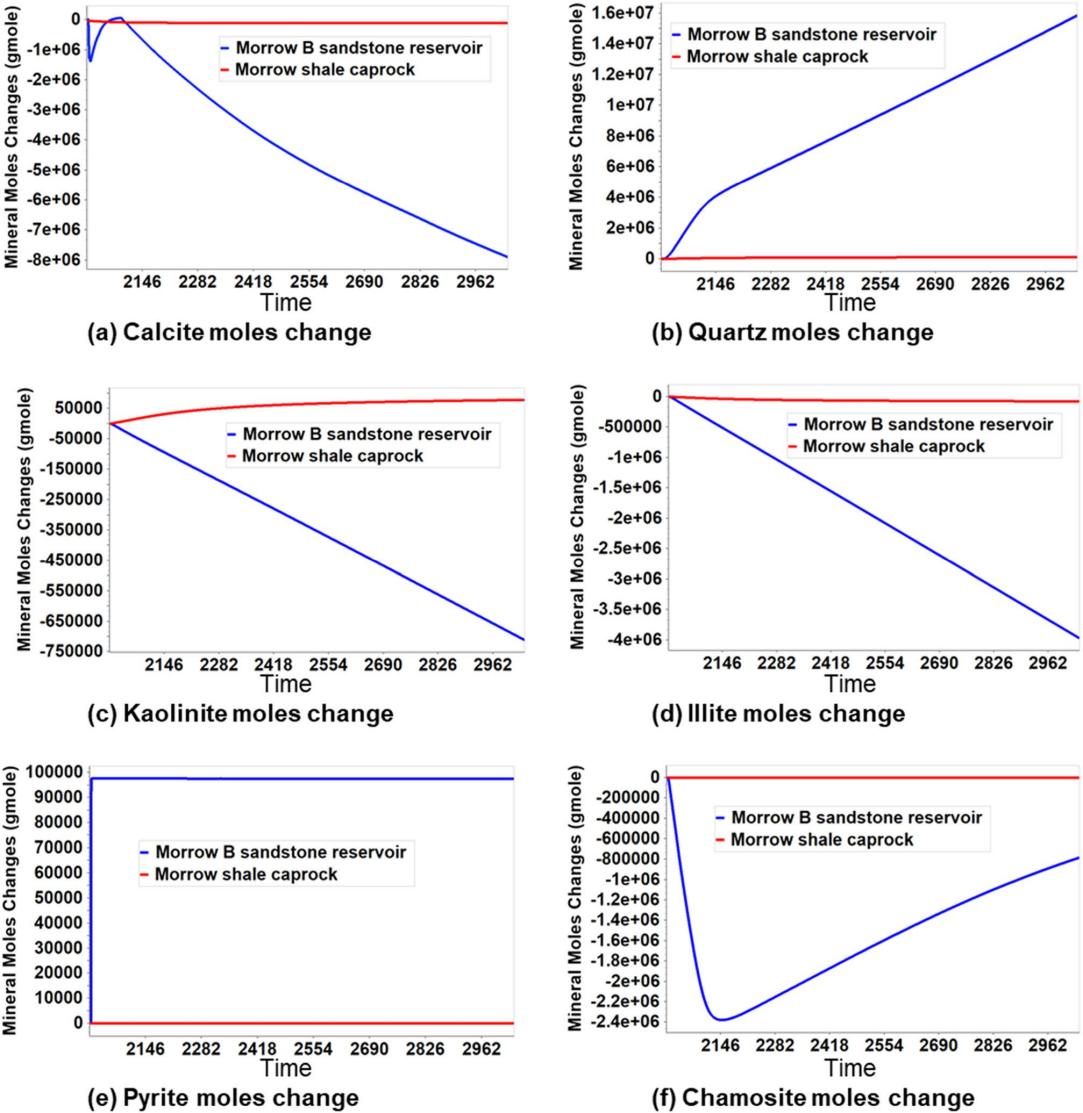
The evolution of dissolution and precipitation of minerals in the reservoir and the caprocks during 1000 years of post-injection monitoring.

The geochemical-induced porosity changes within the caprock and reservoir were studied. The evolution of mineral precipitation and dissolution were the main factors contributing to the geochemical-induced porosity changes as the CO_2_ plume migrates. The coupled hydro-geochemical simulation results indicate that free-phase CO_2_ migrates to the reservoir’s upper layers due to buoyancy or the free-phase CO_2_ being less dense than the resident fluid. As more CO_2_ accumulated at the upper part of the reservoir, relatively more interactions of CO_2_ with the aqueous phase and with formation minerals occurred at the accumulation site than elsewhere. These interactions resulted in the precipitation and/or dissolution of minerals. Figure 7 shows the progression of mineral precipitation and dissolution within the reservoir and the caprock. According to the results, more geochemical reactions occurred within the reservoir than within the caprock because the vast majority of the CO_2_ injected into the reservoir is sealed off by the low permeability adjacent layers of caprock.

**Figure 8:**
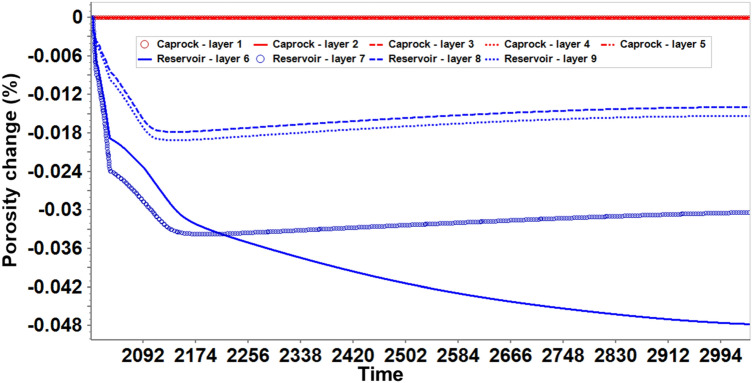
The porosity changes along the injector wellbore grid blocks during 1000 years of post-injection monitoring, showing that the reaction in upper reservoir layers (layer 6 and 7) is more significant than in the bottom reservoir layers (layer 8 and 9); The reaction barely affected the porosity in caprock layers (layer 1 to 5).

**Figure 9:**
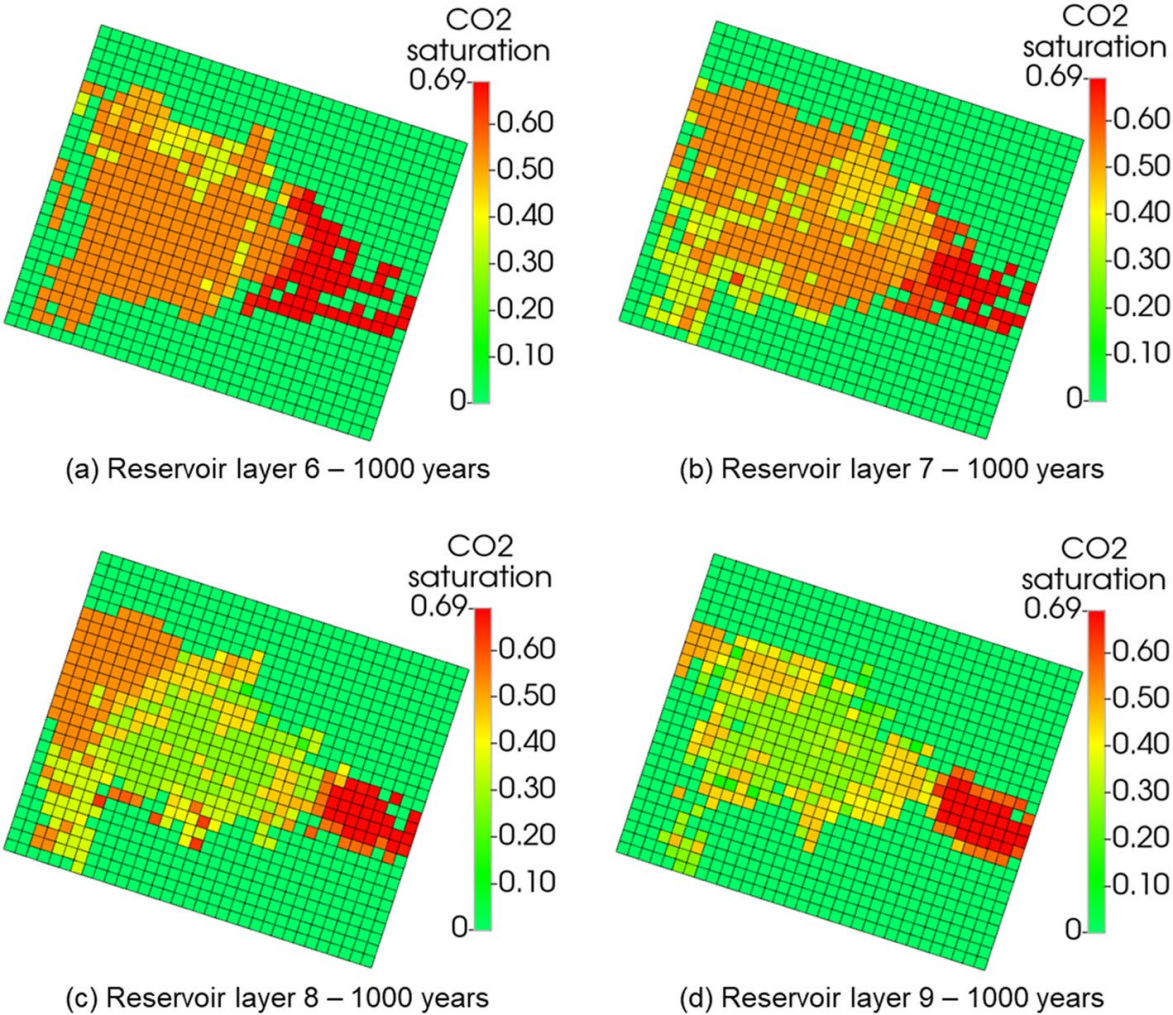
CO_2_ in the reservoir layers at the end of the 1000 years of post-injection monitoring.

**Figure 10:**
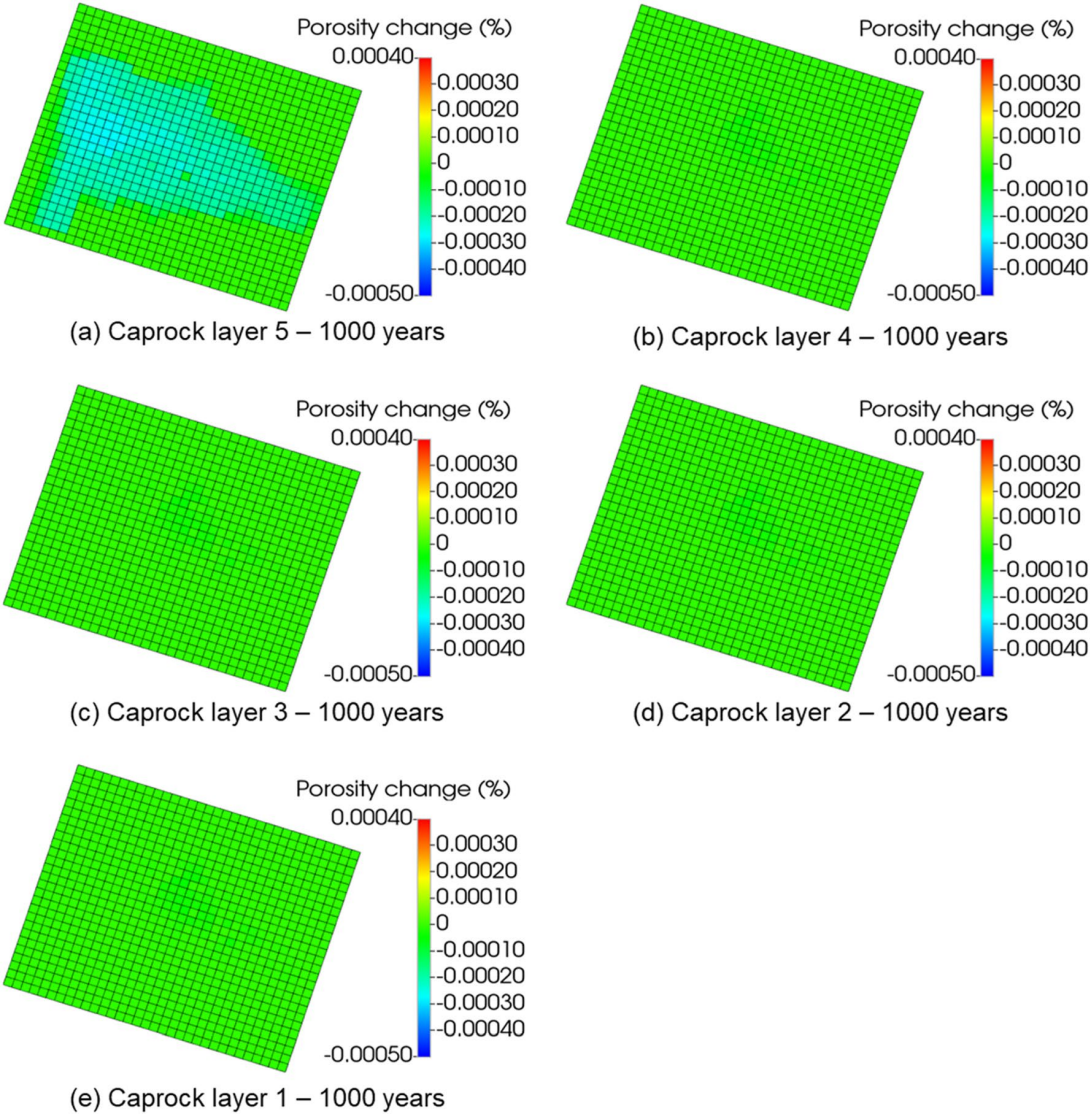
The porosity changes within the caprock layers at the end of the 1000 years of post-injection monitoring.

Further analysis was performed to investigate how far the CO_2_ might migrate upwards and what petrophysical effects would be produced on the caprock by considering the geochemical-induced porosity changes in both reservoir and caprock. Figure 8 shows the porosity changes within the grid block surrounding the injector wellbore in every layer of the reservoir and caprock. Layers 6 and 7, which are the upper part of the reservoir, showed more geochemical activity than the bottom layers, 8 and 9. This is because most CO_2_ migrated to the upper layers (Fig. 9), causing acidification of the reservoir brine, which is favorable for the dissolution of calcite, kaolinite, illite, and chamosite. The dissolution of kaolinite, illite, and chamosite resulted in the release of SiO_2_, which eventually precipitated into quartz. Though the results indicated that the upper layers of the reservoir experienced the largest porosity reduction of about 0.05% (layer 6), all the caprock layers showed a near-zero percent porosity change. This implies that the CO_2_ has negligible geochemical effects on the porosity of the caprock. In probing further, Fig. 10, displays the areal view of the porosity changes in the caprock. Porosity changes occurred mostly in the caprock’s bottom layer (Layer 5). However, the maximum change was about 0.0003% reduction, reinforcing the observation that geochemical reactions did not have significant effects on the caprock. Recent core and thin section analyses have revealed the presence of additional minerals such as siderite, dolomite, and feldspar^31,51^. Therefore, future works should include these minerals.

### Geomechanical response on the caprock due to CO_2_ injection

**Figure 11:**
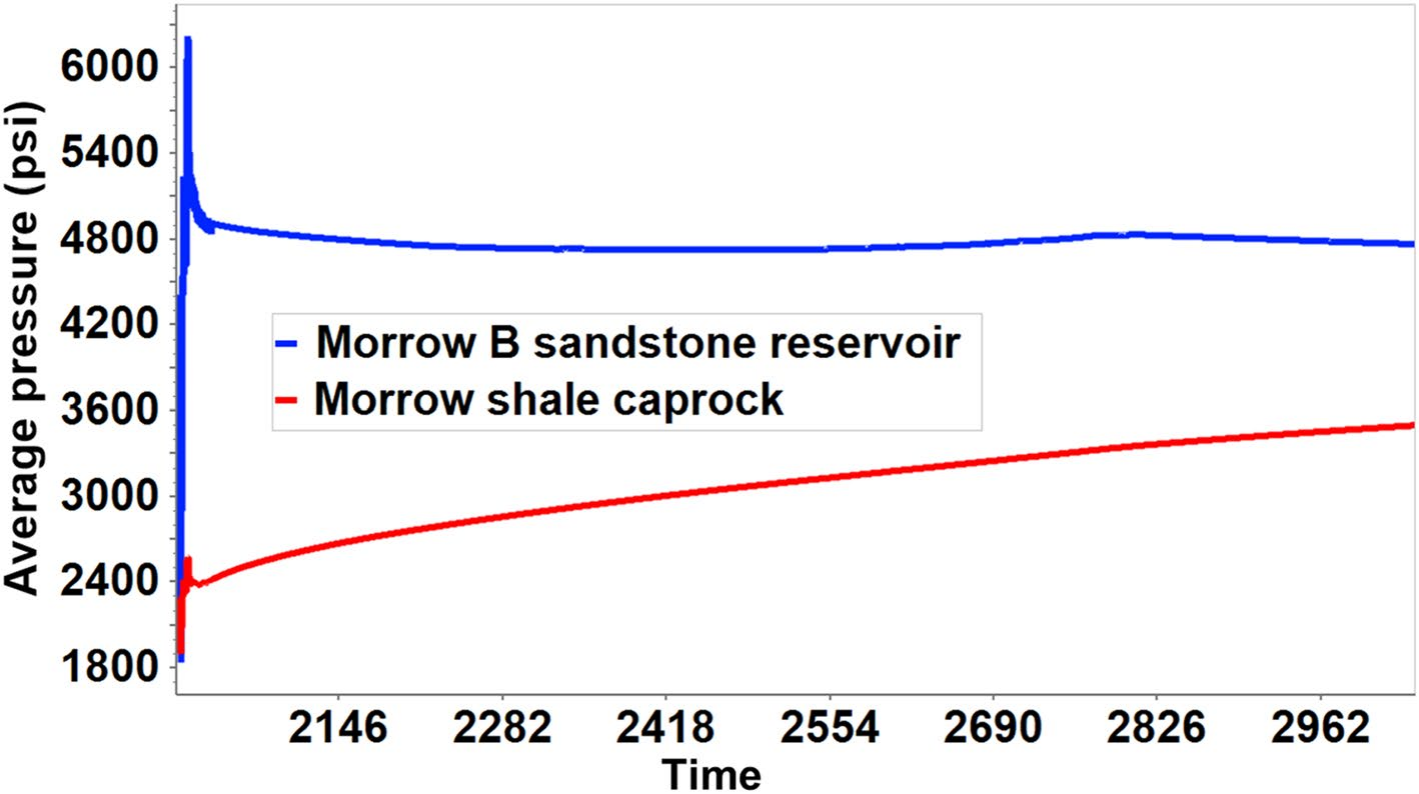
The average pressure within the Morrow B sandstone reservoir and the Morrow shale caprock over time.

**Figure 12:**
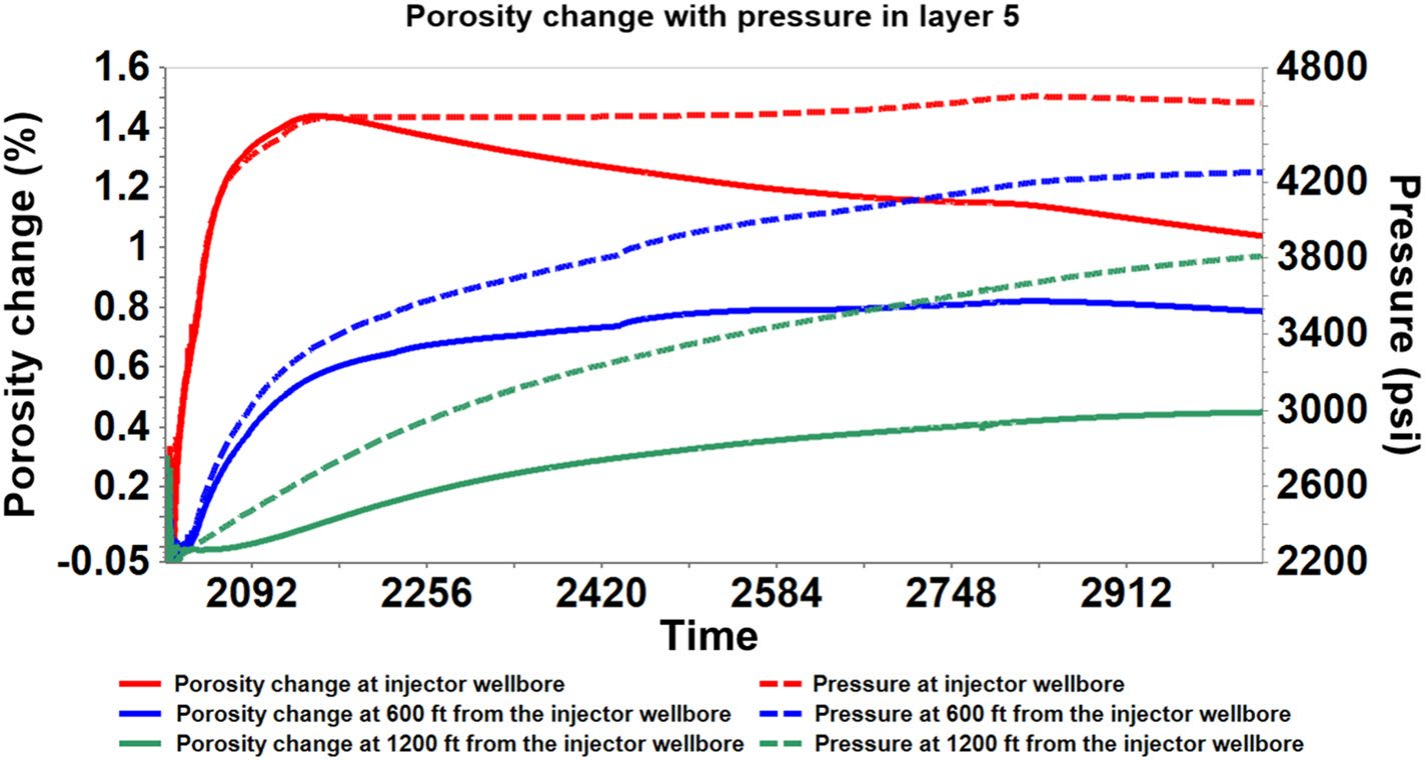
The evolution of stress-induced porosity changes with pressure within the bottom caprock layer (Layer 5) at variable distances from the wellbore during 1000 years of monitoring.

**Figure 13:**
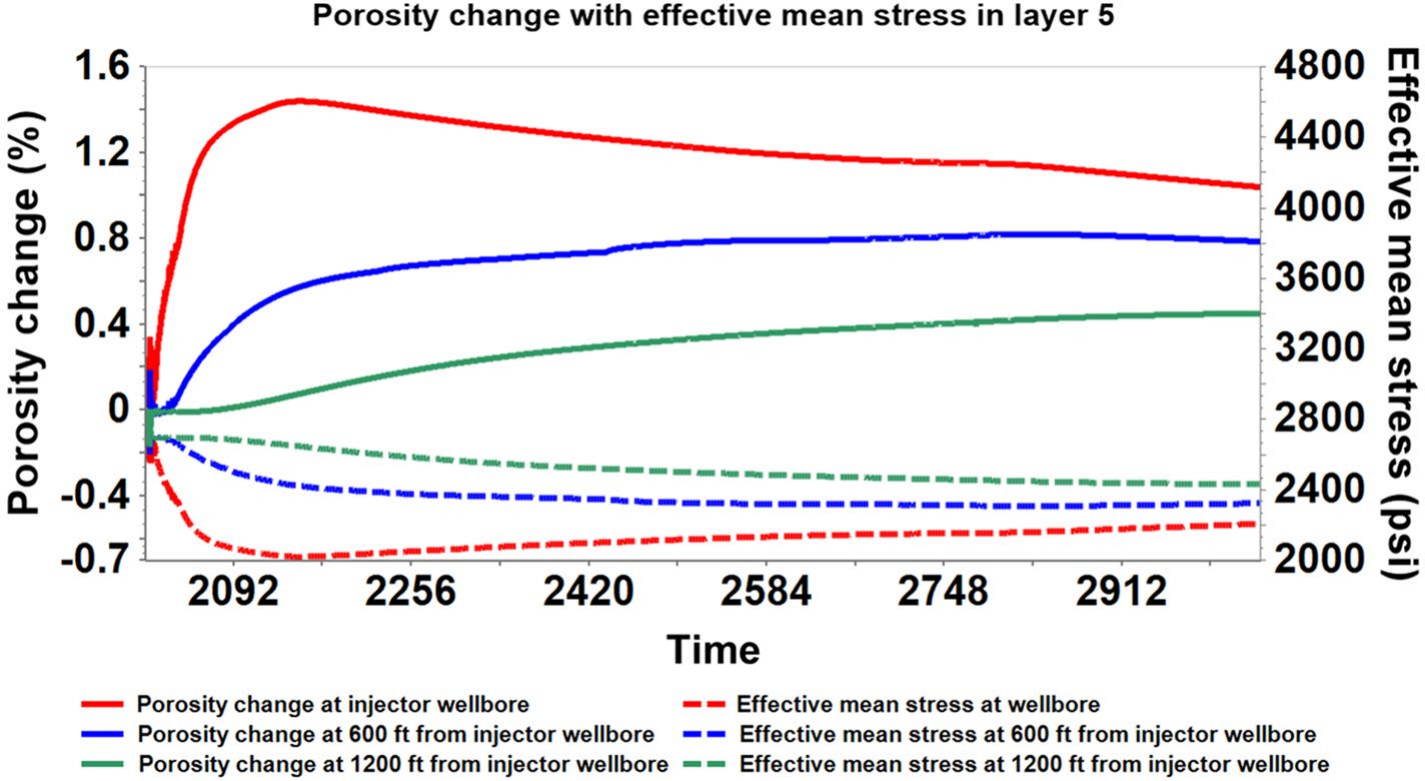
The evolution of stress-induced porosity changes with effective mean stress within the bottom caprock layer (Layer 5) at variable distances from the wellbore during 1000 years of post-injection monitoring.

**Figure 14:**
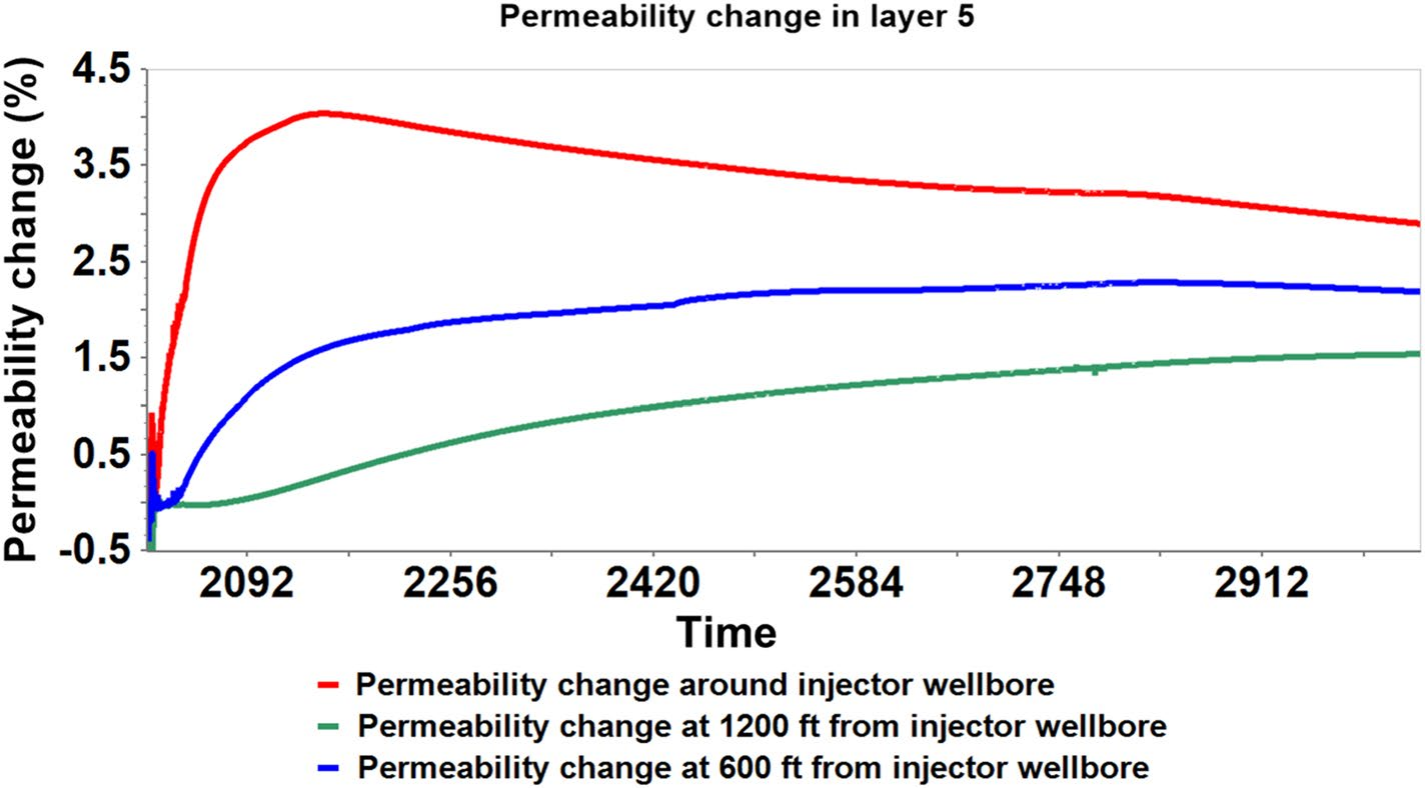
The evolution of stress-induced permeability changes within the bottom caprock layer (Layer 5) at variable distances from the wellbore during 1000 years of post-injection monitoring.

Utilizing the coupled hydro-geomechanical simulation results, the porosity and permeability properties alterations were analyzed. The stress-induced porosity is a function of temperature, pressure, and total mean stress; however, the model was run assuming isothermal conditions; therefore, only pressure and total mean stress were used to estimate the porosity. After attaining a maximum average reservoir pressure (6227 psi) in 2020–02, there was a gradual rise in the caprock pressure as observed in Figs. 11 and 12. Concurrently, there was a gradual decrease in effective mean stress and an increase in porosity in the bottom layer of the caprock, according to Fig. 13. Changes in pressure, effective mean stress, and porosity all decay with distance away from the wellbore. The increase in porosity is attributed to the slow migration of CO_2_ from the reservoir to the bottom layer of the caprock that causing an expansion of the pore volume. In addition, permeability, which is almost always proportional to the porosity, increased due to the increase in effective mean stress and vice versa. Figure 14 shows that the permeability increased with an increase in effective mean stress but decays off with distance away from the injector wellbore. The maximum porosity and permeability changes were approximately 1.4% and 4% respectively.

**Figure 15:**
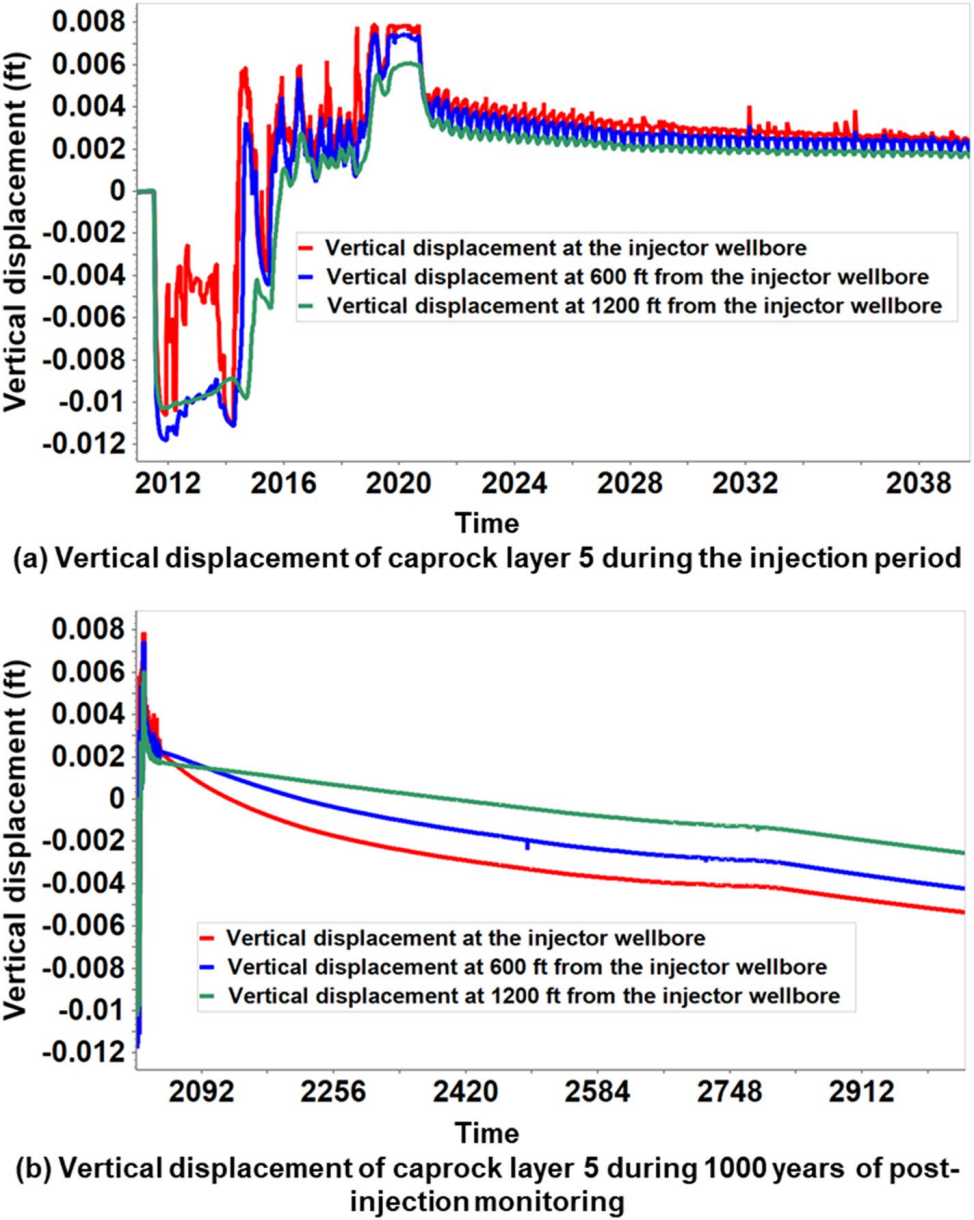
Evolution of vertical displacement of caprock layer 5.

The subsidence and uplift of the caprock and reservoir pose risks to CO_2_ sequestration operations. The vertical displacement was considered to analyze the subsidence and uplift of the caprock. Figure 15 displays the vertical displacement of the caprock at the wellbore, 600 ft and 1200 ft away from the wellbore. Subsidence may occur due to the contraction of the pore space as a result of the reservoir pressure reduction. On the contrary, uplift occurs when the reservoir pressure increases and the pore volume expands. In 2019 and 2020, the injection pressure in the reservoir increased and caused an expansion of the pore space, which resulted in an uplift of 0.008 ft (about 2.4 mm) of the caprock around the wellbore (Fig. 15A). Subsequently, when the injection pressure was cut back to 5500 psi after 2020, this was reflected in a 0.003 ft (about 0.9 mm) reduction of the uplift. The subsidence in the caprock (Fig. 15B) gradually increased when the reservoir was monitored for 1000 years due to some migration of CO_2_ into the bottom layer of the caprock.

**Figure 16:**
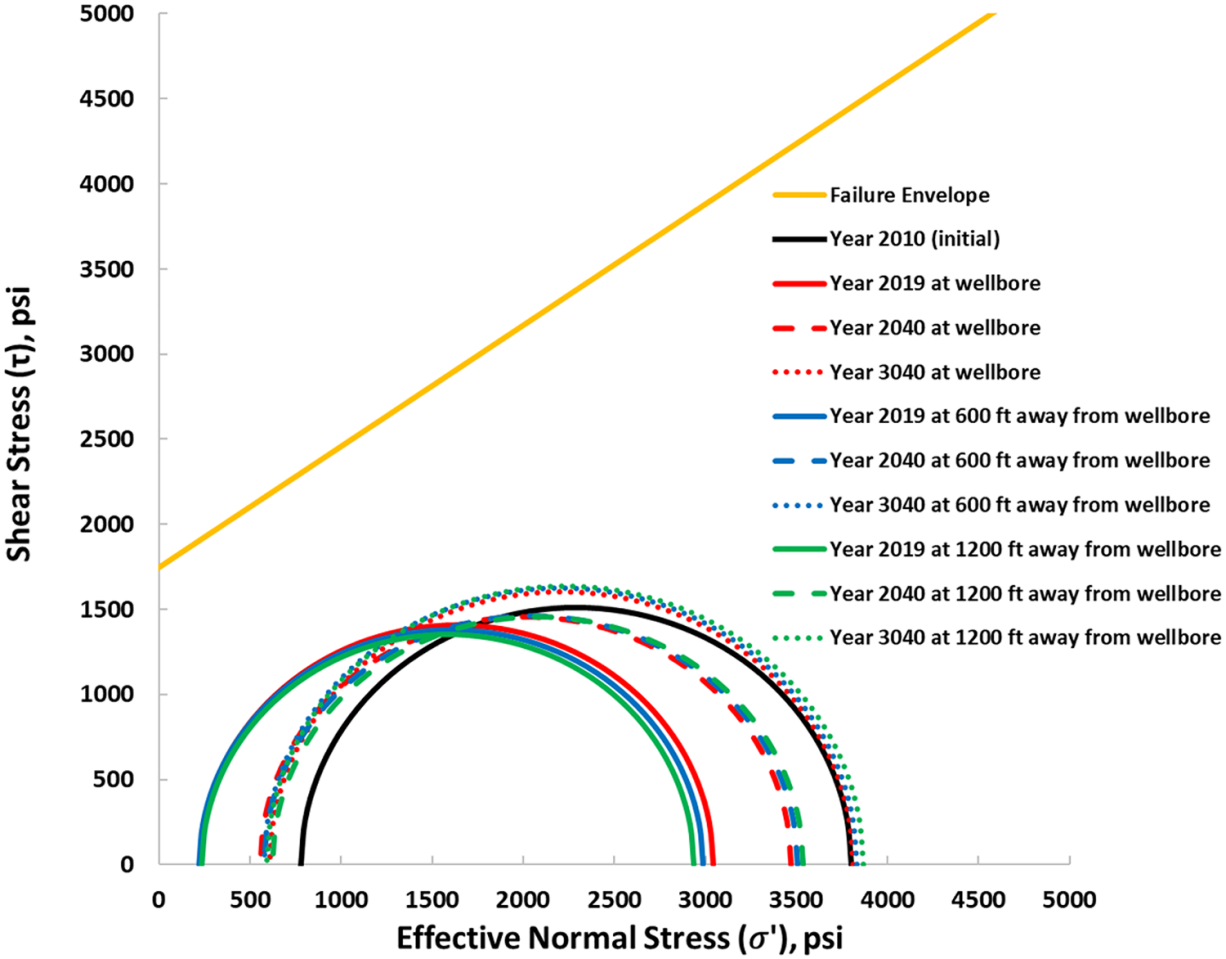
Mohr circles depicting the stress changes within the reservoir (Layer 6) when 5500 psi BHP injection pressure was used.

**Figure 17:**
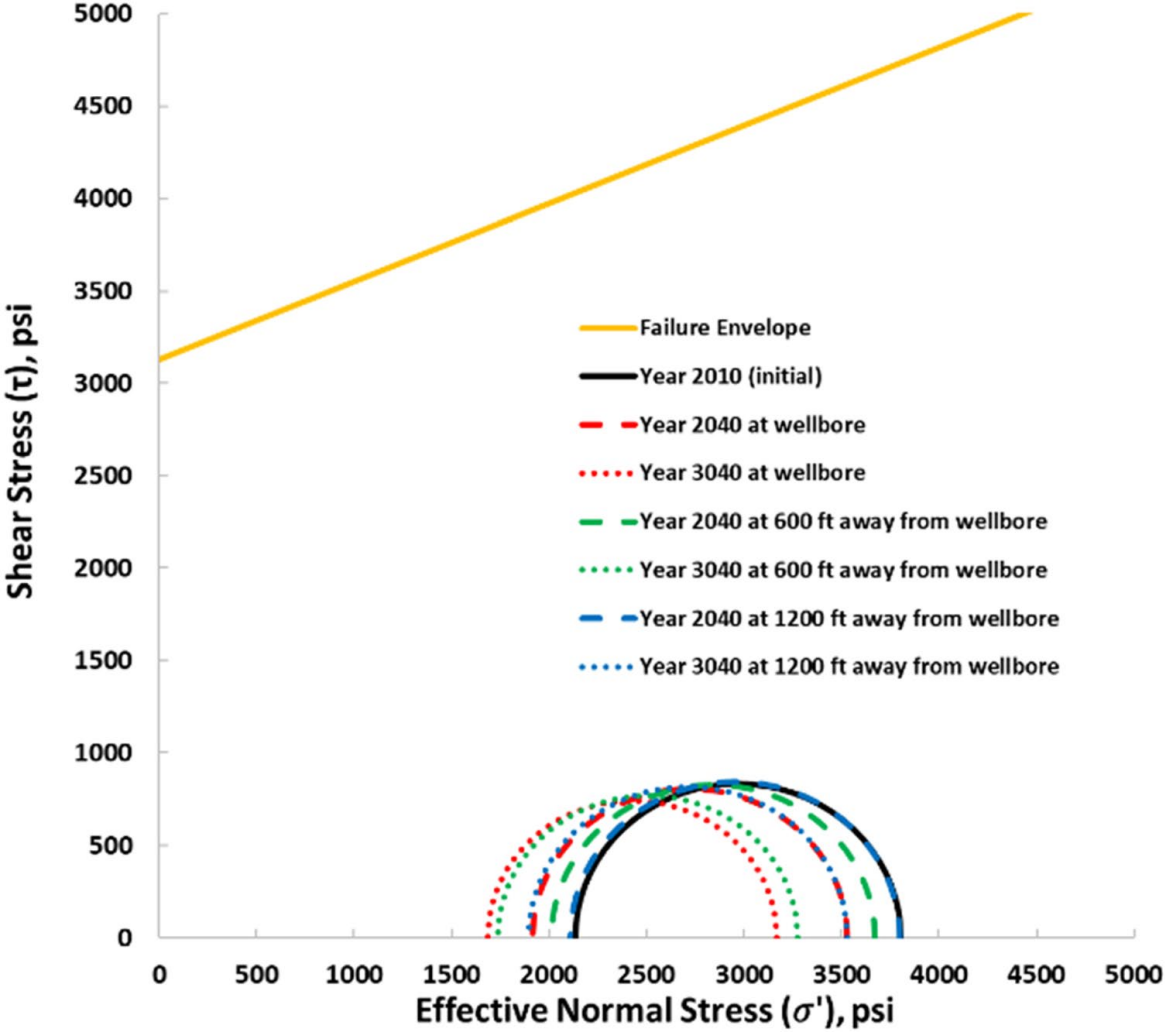
Mohr circles depicting the stress changes within the caprock (Layer 5) when 5500 psi BHP injection pressure was used.

The Mohr–Coulomb failure and the shear safety factor were analyzed at the reservoir–caprock interface to confirm the overall long-term safety of the caprock. Figures 16 and 17 show the Mohr circles illustrating the stress behavior at the reservoir–caprock interface at different distances from the wellbore and at different time intervals. The black Mohr circle is the initial condition before the tertiary recovery phase in 2010. The circles move to the left (lower effective stress) the circle's size also decreases (Fig. 16) as the pore pressure increases within Layer 6 of the reservoir. The initial average pore pressure in 2010 was about 4390 psi, which increased to an average pore pressure of 6224 psi in 2019. Similarly, as the pore pressure decreases, the circles move to the right (higher effective stress) and increase in size. Thus, even though the Mohr circles enlarge at the end of the 1000 years of monitoring, they do not touch the failure line. Also, within the caprock (Layer 5), as indicated in Fig. 17, the generated Mohr circles shifted to the left as the pore pressure gradually increased, implying lower effective stresses within Layer 5. However, the Mohr circles shown in the caprock are far from failure as they are far below the failure envelope.

**Figure 18:**
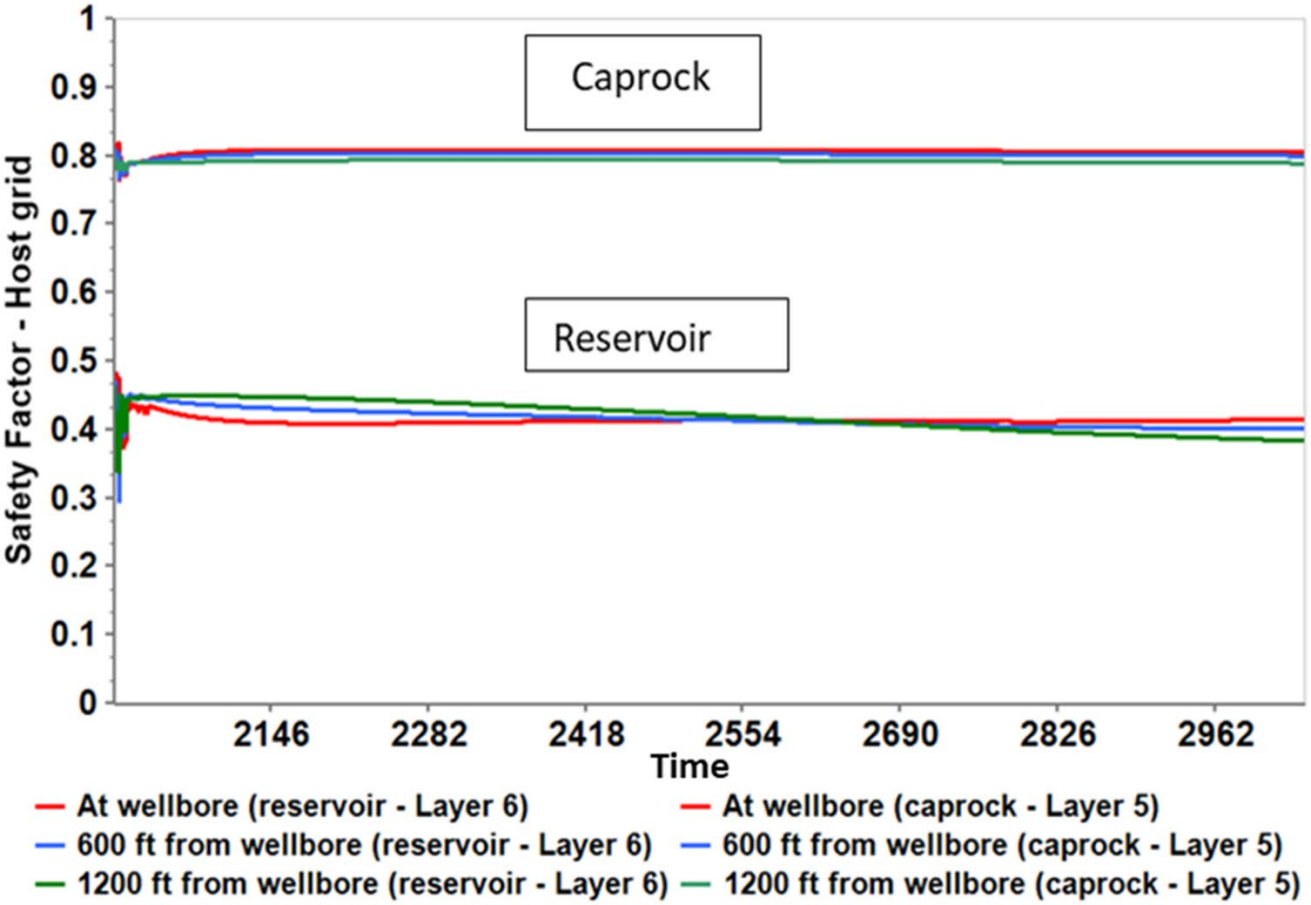
Shear Safety Factor at the reservoir and caprock.

The Mohr circles and the failure envelopes in Figs. 16 and 17 were also expressed as shear safety factors corresponding to various distances from the wellbore in Fig. 17. A safety factor of zero indicates that the Mohr circle touches the failure envelope, so the rock fails. In Fig. 18, the safety factors for the reservoir and caprock are above 0.3 and 0.7, respectively. In other words, the reservoir and caprock remained elastic till the end of the 1000-year post-injection monitoring period. The integrity of the caprock can be validated with the work of Trujillo^52^, who determined that the upper Morrow shale caprock has an excellent seal capacity as the minimum CO_2_ column height was 3609 ft which was more than the values suggested by Gibson-Poole et al.^53^. Again, Trujillo^52^ determined that the upper Morrow shale mechanical properties, low values of Young’s modulus together with the high values of Poisson’s ratio, made it a more ductile formation than the reservoir. Lastly, Trujillo^52^ estimated the maximum pore pressure of 6518 psi at the reservoir and caprock interface below which the caprock would maintain its mechanical stability.

### The long-term CO_2_ storage capacity of the FWU

**Figure 19:**
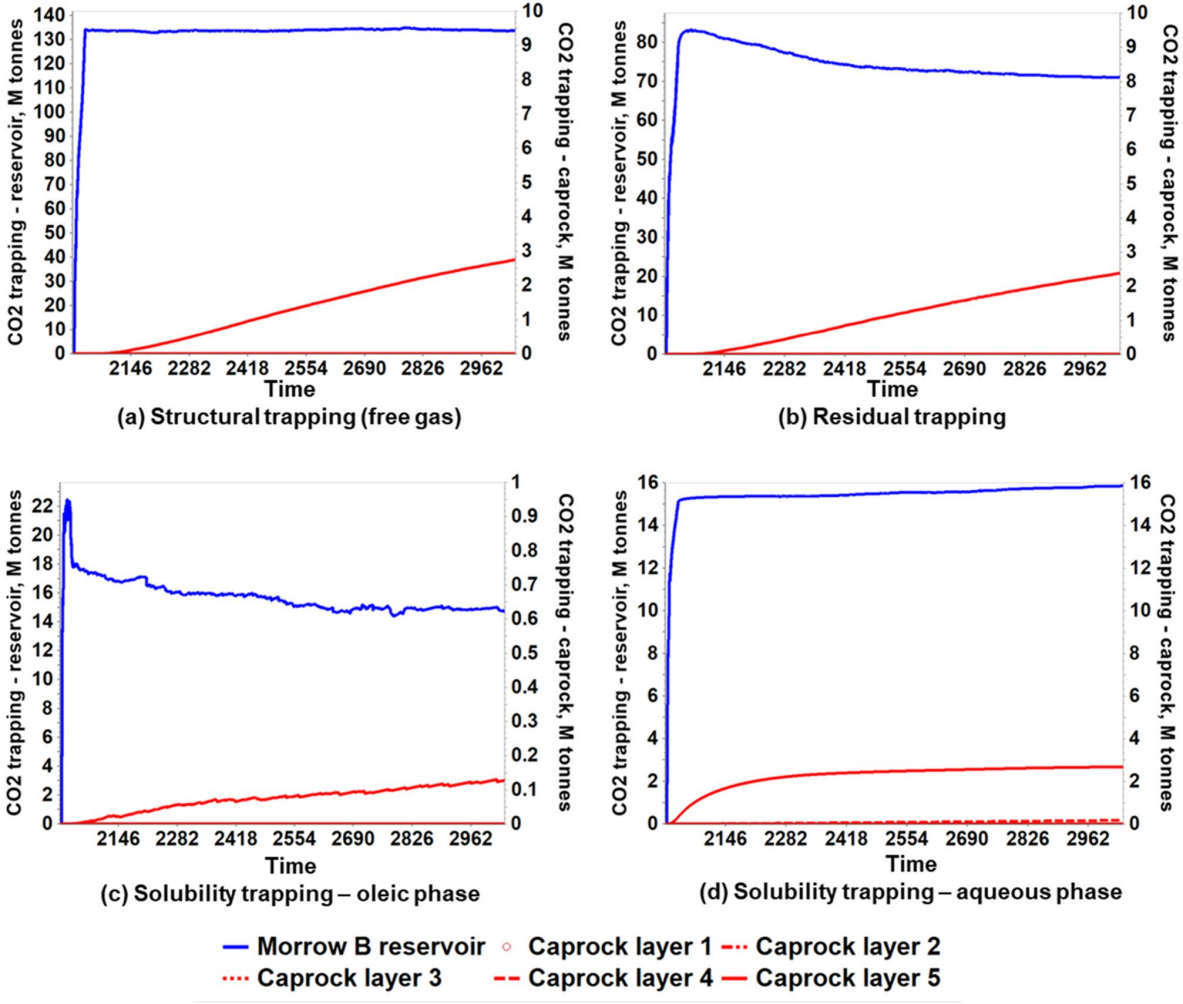
The evolution of CO_2_ storage distribution in reservoir and caprock.

As the caprock was determined to be a valid long-term seal according to the Mohr–Coulomb circles and the safety factors, the effects of the porosity and permeability alterations on the reservoir storage mechanisms were analyzed. In this analysis, the amount of CO_2_ contained within the Morrow B reservoir and the amount migrated into the caprock over the monitoring period of 1000 years were considered to ascertain the long-term storage capacity of the FWU. Historically, CO_2_ was injected into the 13-10A pattern at variable bottom-hole pressures from 2014 until 2020. Then, additional forecasting was simulated with a baseline BHP constraint of 5500 psi for 20 years. Next, all the wells, production, and injection, were shut in to monitor the CO_2_ plume for an additional 1000 years. Table 5 and Fig. 19 present the simulation results regarding the amount of CO_2_ storage in the reservoir and caprock. Overall, 243.73 M tonnes of CO_2_ were stored within the 13-10A pattern. However, at the end of 1000 years of post-injection monitoring, the Morrow B reservoir retained 235.60 M tonnes of CO_2_, making 96.67% of the stored CO_2_. The amount of CO_2_ migrated into the upper Morrow shale caprock was 7.95 M tonnes, representing 3.33%. The main reason for the CO_2_ migration, even though the caprock was intact according to Mohr–Coulomb failure criteria, is pore space expansion causing a slight increase in porosity and permeability. It was observed that the migrated CO_2_ was mostly retained in the bottom layer (Layer 5) of the caprock, which indicates that the caprock will be able to provide an excellent seal even beyond 1000 years.

**Table 5:** CO_2_ trapped in reservoir and caprock after 1000 years of monitoring with baseline BHP forecasting strategy.

	CO_2_ Trapped, M tonnes					Percentage storage (%)
	Residual	Structural (free gas)	Solubility			
			Aqueous phase	Oleic phase	Total Storage	
**Morrow Shale Caprock**						
Layer 1	0.00	0.00	0.00	0.00	0.00	3.33
Layer 2	0.00	0.00	0.00	0.00	0.00	
Layer 3	0.00	0.00	0.00	0.00	0.00	
Layer 4	0.00	0.00	0.18	0.00	0.18	
Layer 5	2.39	2.75	2.68	0.13	7.95	
**Morrow B Reservoir**						
Layers 6 to 8	71.13	133.88	15.86	14.74	235.60	96.67
Total Storage	73.51	136.63	18.72	14.87	243.73

**Figure 20:**
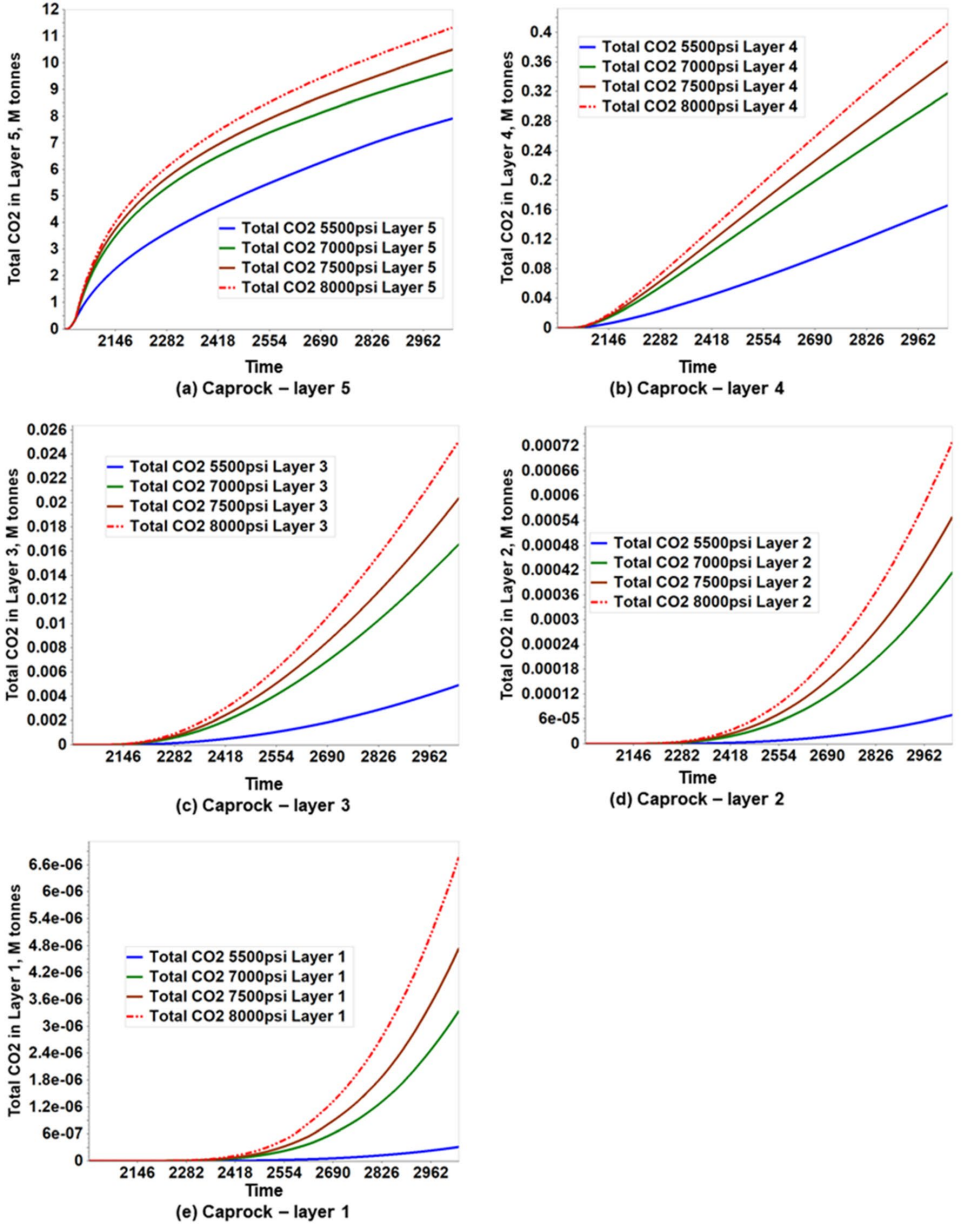
Amount of CO_2_ accumulated in the caprock layers resulting from pressure sensitivity analysis.

**Figure 21:**
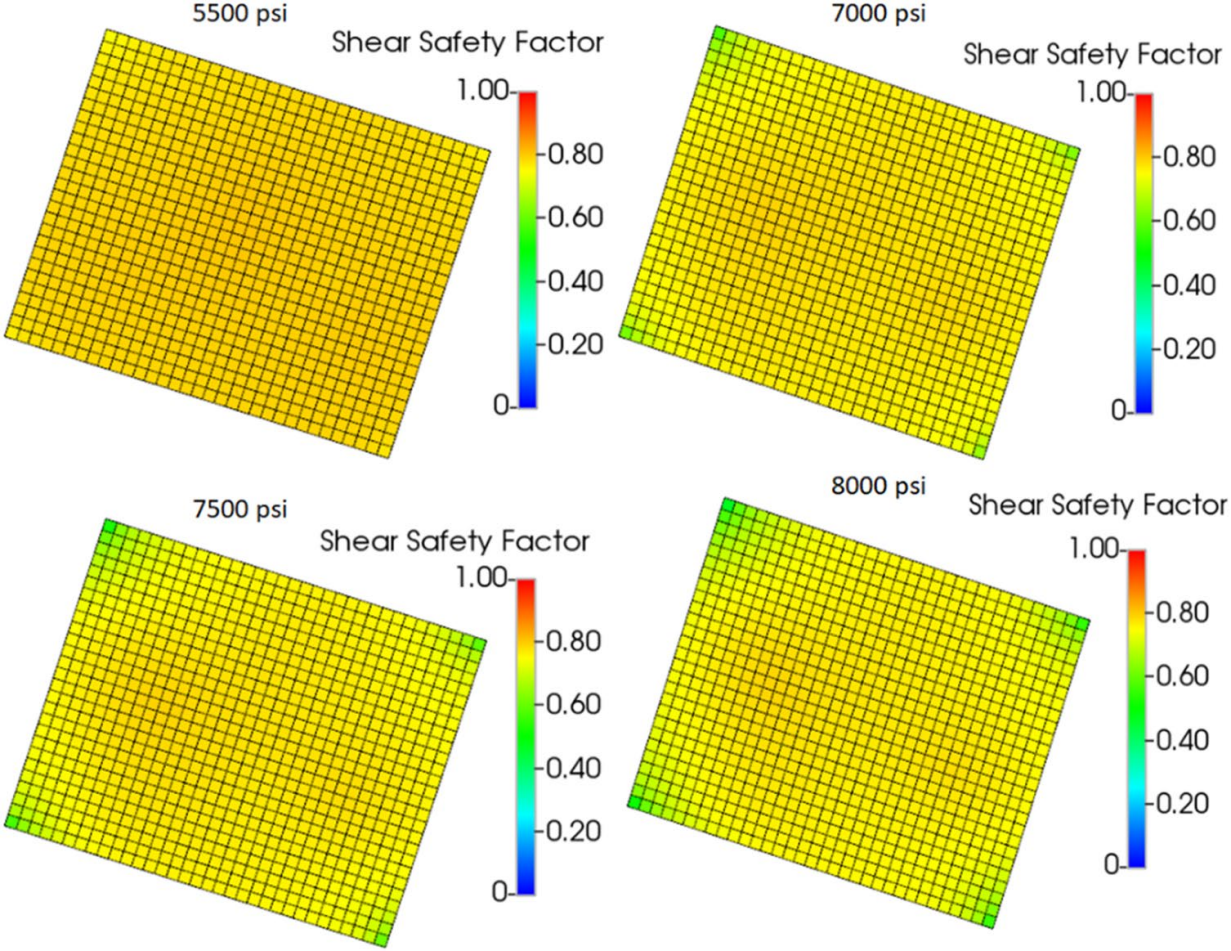
Shear safety factor in the bottom layer (Layer 5) of the caprock.

Figures 20 and 21 show the amount of CO_2_ accumulated in the caprock layers and the shear safety factor of the caprock’s entire bottom layer (Layer 5), respectively, according to the pressure sensitivity analysis performed. According to Fig. 20, more CO_2_ migrates to the caprock layers by increasing the injection BHP constraint. This is because as BHP increases, more CO_2_ is injected, causing high pore pressure that favors increased fluid migration. With all the BHP constraints (5500 psi, 7000 psi, 7500 psi, and 8000 psi) used, Layer 5 retained the most CO_2_. Also, a substantial amount of CO_2_ migrated into Layer 5. The risks to the caprock associated with each BHP constraint were indicated in Fig. 21. Despite the fluid migration in the sensitivity analysis, the shear safety factor suggests that the caprock remains intact as it remains above 0.3 at all locations, which means that the caprock is mechanically stable.

### Implication and limitations

This study aimed to ascertain the effects of chemo-mechanical processes on the upper Morrow shale caprock in the long-term post-injection of CO_2_ into the reservoir to determine the caprock integrity and the long-term storage capacity of the FWU. In this study, a coupled hydro-geochemical simulation model was utilized to examine the impact of the geochemical processes, such as the dissolution and precipitation of the caprock minerals and the resultant impacts on porosity. In addition, a coupled hydro-geomechanical simulation model was employed to assess the impacts of stress and pressure changes on porosity, permeability, ground displacement, and the extent of CO_2_ migration.

On the topic of caprock integrity of field scale CCUS project. The study has been mostly focused on the geomechanical impact as the significant amount of CO_2_ will fluctuate the connate stresses in the storage reservoir as well as the caprock formations above. Many more geochemistry studies investigated the rock mineralizations that occurred in the storage reservoir other than the potential weakness uncertainty that may be induced by the reactions that occurred between formation fluid and caprock minerals. The process and results demonstrated in this work embodied the significance of considering the impact on caprock integrity from both geomechanics and geochemistry. It brings crucial insights to the industry for the wellness of a large-scale CO_2_ storage project.

In spite of the fact that the analysis performed in this case is justified, some important assumptions can be improved in future works to bolster the study at the current stage. First of all, The current work used an empirically determined stress-permeability relationship by Touhidi-Baghini^54^ to estimate stress-induced permeability. In the absence of stress-permeability experimental data available, the empirical method was utilized. However, using experimentally determined stress-permeability tables from the field rock samples would improve the accuracy of stress-induced permeability. Secondly, The current study modeled the six minerals determined by ELAN analysis as primary minerals within the formation. However, the experimental works done on thin sections have revealed the presence of other minerals. Therefore, it would be beneficial to model secondary mineral reactions to account for any precipitation of such secondary minerals. Lastly, Since coupling multidisciplinary models, hydrodynamical, geochemical, and geomechanical, results in a computationally complex model, running such a model on a full-field scale will require more computational resources. Coupled hydro-geo-chemical-mechanical model on a full-field scale would be beneficial in accurately estimating the CO_2_ sequestered within the entire Farnsworth field unit or any other field scale CCUS projects.

## Conclusion

Based on the results and analysis of this study, the following conclusions are made:

1. The precipitation and dissolution of the minerals at the reservoir–caprock interface had insignificant effects on porosity. This is because the maximum porosity change estimated around the wellbore within the caprock was approximately 0.0003%. The negligible impact of geochemical reactions on porosity may result from the lack of highly reactive minerals.
2. The coupled hydro-geomechanical model's effective mean stress and pressure changes accounted for a more significant influence on porosity and permeability. Furthermore, these effects were associated with pore space expansion; the maximum porosity and permeability changes were estimated at 1.4% and 4%, respectively.
3. According to the Mohr–Coulomb failure criteria, the caprock is far from a failure as the computed shear safety factor was above 0.7. Therefore, the rock material was elastic during the entire simulation period. In addition, pressure sensitivity analysis indicated that the caprock will still be intact and free from shear failure at an injection pressure of 8000 psi.
4. 96.7% of the sequestered CO_2_ remained in the Morrow B reservoir, and only 3.3% migrated to the caprock after 1026 years. Although about 3.3% of the stored CO_2_ migrated into the caprock, the seal integrity of the caprock is still good, as almost all the CO_2_ migration was confined in the bottom layer of the five-layer caprock.
5. Finally, the geochemical and geomechanical analysis suggests that the upper Morrow shale caprock is both chemically and mechanically stable to seal off the CO_2_ sequestrated in the Morrow B sandstone reservoir.

## Data Availability

The raw production and injection dataset that support the findings of this study are available from the operator of the Farnsworth Unit, Perdure Petroleum, LLC but restrictions apply to the availability of these data as it is considered as confidential property of the operator.

## List of symbols

$$C$$: Land’s constant
$Sgm$: Maximum gas saturation
$Sgcrit$: Critical gas saturation
$K_{rg}^{drain}$: Relative permeability estimated for the drainage scanning curve
$$S_{g}$$: Gas saturation
$S_{w}^{start}$: Water saturation at the beginning of the secondary drainage
$K_{rg}^{input}$: Input relative permeability at $S_{g}$
$K_{rg}^{input} \left( {S_{g}^{start} } \right)$: Input relative permeability at saturation at the start of the secondary drainage curve
$K_{rg}^{imb} \left( {S_{g}^{start} } \right)$: Relative permeability at the beginning of the secondary drainage
$S_{wcon}$: Connate water saturation
$$\alpha$$: Reduction exponent
$f_{co2,g}$: Fugacity of CO_2_ in the gaseous phase
$f_{co2,aq}$: Fugacity of CO_2_ in the aqueous phase
$H_{co2,aq}$: Henry’s law constant
$y_{co2,aq}$: Molar fraction of CO_2_ dissolved in the aqueous phase
$r_{\theta }$: Rate of the reaction
$\hat{A}_{\theta }$: Reactive surface area of mineral $\theta$
$k_{\theta }$: Rate constant for mineral $\theta$
$Q_{\theta }$: Activity product of mineral reaction $\theta$
$K_{eq,\theta }$: Chemical equilibrium constant for mineral reaction $\theta$
$R_{mn}$: Number of mineral reactions
$\phi_{res}^{n + 1}$: Reservoir porosity at $n + 1$ timestep
$V_{b}^{0}$: Initial bulk volume
$$V_{p}$$: Pore volume
$$\alpha_{b}$$: Biot’s constant
$\sigma_{m}$: Mean total stress
$\beta$: Volumetric thermal-expansion coefficient
$$E$$: Young’s modulus
*Γ*: Coupling factor
$$v$$: Piosson’s ratio
$$c_{b}$$: Bulk compressibility
$c_{r}$: Solid rock compressibility
$\varphi$: Internal friction angle
$$C$$: Cohesion
